# Investigation of cellulose dissolution in morpholinium-based solvents: impact of solvent structural features on cellulose dissolution†

**DOI:** 10.1039/d3ra03370h

**Published:** 2023-06-20

**Authors:** Shirin Naserifar, Andreas Koschella, Thomas Heinze, Diana Bernin, Merima Hasani

**Affiliations:** a Department of Chemistry and Chemical Engineering, Chalmers University of Technology 412 96 Gothenburg Sweden shirinn@chalmers.se +46317722999; b Wallenberg Wood Science Center, Chalmers University of Technology 412 96 Gothenburg Sweden; c Center of Excellence for Polysaccharide Research, Institute of Organic Chemistry and Macromolecular Chemistry, Friedrich Schiller University of Jena Humboldtstraße 10 07743 Jena Germany

## Abstract

A series of *N*-methylmorpholinium salts with varying *N*-alkyl chains and Cl^−^, OAc^−^ and OH^−^ as counter ions have been synthesized and investigated for their ability to dissolve cellulose, aiming at elucidating solvent structural features affecting cellulose dissolution. Synthesis procedures have been developed to, to a high extent, rely on conversions in water and microwave-assisted reactions employing a reduced number of work-up steps and ion-exchange resins that can be regenerated. Water solutions of morpholinium hydroxides proved capable of dissolving cellulose, with those of them possessing alkyl chains longer than ethyl showing surprising dissolution ability at room-temperature. Morpholinium acetates behaved as ionic liquids, and were also capable of dissolving cellulose when combined with DMSO. The obtained cellulose solutions were characterized according to their chemical and colloidal stability using ^13^C NMR spectroscopy, size exclusion chromatography and flow sweep measurements, while the ethanol coagulates were investigated in terms of crystallinity using solid state NMR. In contrast, the morpholinium chlorides obtained were hygroscopic with high melting points and low solubility in common organic solvents *e.g.*, acetone, DMSO and DMAc, thus lacking the ability to swell or dissolve cellulose.

## Abstract 2

A series of *N*-methylmorpholinium salts with varying *N*-alkyl chains and Cl^−^, OAc^−^ and OH^−^ as counter ions have been investigated for their ability to dissolve cellulose aiming at elucidating solvent structural features affecting cellulose dissolution.

## Graphical abstract

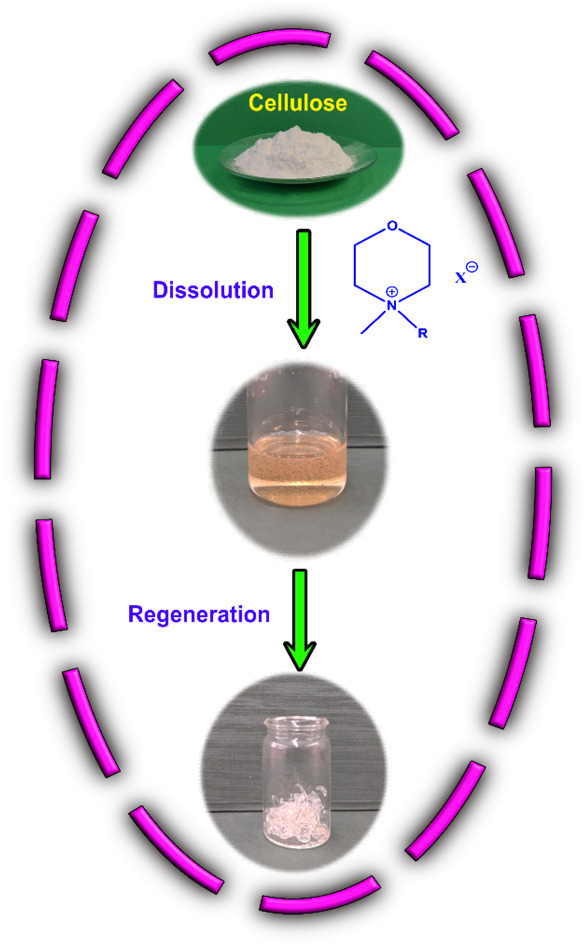

## Introduction

Cellulose, being the most abundant biopolymer on Earth, occurs naturally as the main structural polymer of the cell wall in higher plants as well as in algae, tunicate and bacteria. Since it is biodegradable, biocompatible and possesses thermal and chemical stability,^1,2^ it has been widely used in a range of products such as pulp, paper, cardboard, textiles, films, cellulose derivatives,^3^*etc.* However, cellulose does not melt^4^ as its melting point is higher than its degradation temperature.^5^ Consequently, one critical prerequisite for the processing of this important polysaccharide (*e.g.*, shaping and chemical modification) is its dissolution – a highly challenging process due to the extensively stabilized hierarchical organization of cellulose. Equatorially positioned hydroxyl groups in a cellulose chain promote, both intramolecular H-bonding within the chain as well as intermolecular H-bonding between the neighboring chains. The former leads to flat chain conformation, stiffness, and the tendency to crystallize while the latter stabilizes a sheet-like structure and promotes stacking of the sheets on top of each other through exclusion of water and van der Waals interactions (the absence of hydroxyl groups creates more hydrophobic regions in the axial direction). Thus, cellulose is amphiphilic and has highly ordered supramolecular structure extending to organization in semicrystalline fibrils. These fibrils are further arranged in a layered hierarchical morphology of a cellulose fiber making it largely insoluble in the most commonly used solvents. Hence, the main challenge is to develop a solvent capable of overcoming the mentioned amphiphilicity and preventing attractive interactions in a dissolved state.

In this regard, two dissolution systems have been of particular commercial importance. In viscose process, CS_2_/NaOH(aq) is used as a derivatizing solvent in which cellulose is transformed to NaOH(aq) soluble cellulose xanthate. However, CS_2_ is toxic and flammable polluting agent. In lyocell process, *N*-methylmorpholinium-*N*-oxide (NMMO) monohydrate which is non-toxic, fully biodegradable and fully recyclable is being used.^6,7^ But this solvent is prone to exothermic and oxidative reactions since it is a strong oxidant and is sensitive to alkylating and acylating agents and redox-active transition metal ions.^8^ Development of ionic liquids capable of dissolving cellulose^9–11^ should also be mentioned in this context as it has opened new avenues for shaping and modifying cellulose,^12,13^ with the recycling of the solvent remaining as one of the main challenges. *N*,*N*-dimethylacetamide (DMAc)/LiCl is another well-studied solvent mainly used in gel permeation chromatography (GPC) for evaluating molecular weight distribution of cellulose^14^ as well as for its homogeneous functionalization. Yet, since traces of water or impurities hamper its potential for cellulose dissolution, its application in industrial scale has not been possible.^15,16^ Therefore, there is a need to overcome the aforementioned setbacks with the already existing solvents and promote sustainable processes which makes aqueous cellulose solvents particularly attractive.

In early 1930s, Davidson discovered that NaOH(aq) is able to dissolve cellulose in limited range of concentrations and at low temperatures.^17,18^ However, most of the untreated cellulose showed partial dissolution. Later on, application of different additives such as ZnO, urea, thiourea and polyethylene glycol was evaluated aiming at dissolution enhancement.^19–23^ Even though NaOH is inexpensive and easily recycled, limited solvent concentration range needed for cellulose dissolution, required low temperature, low dissolution capacity for high-molecular weight cellulose and, instability of solutions over time remain as major disadvantages of this system.^17,24,25^ Yet, another group of aqueous hydroxide solvents that has been the subject of extensive studies is quaternary ammonium hydroxides, initially patented by Lilienfeld.^26^ Later, a number of quaternary ammonium hydroxides (QAHs) were reported to be capable of dissolving cellulose, namely, tetramethylammonium hydroxide (TMAH),^27^ tetraethylammonium hydroxide (TEAH),^28^ tetrabutylammonium hydroxide (TBAH),^29^ benzyltrimethylammonium hydroxide (Triton B) and dimethylbenzylammonium hydroxide (Triton F).^30,31^ Apart from hydroxides, a few other anions were combined with QAs, some of combinations showing a promising dissolution ability, such as tetrabutylammonium fluoride (TBAF)/DMSO,^32^ tetrabutylammonium acetate (TBAOAc)/DMSO,^33^ triethylmethylammonium carboxylates,^34,35^ tetraalkylammonium trialkylphosphates^36,37^ and tetraalkylammonium chlorides as well as their mixture with DMA.^38^

Inspired by the NMMO, while striving for water-based hydroxides, our group developed a new aqueous morpholinium-based hydroxide solvent, *N*,*N*-dimethylmorpholinium hydroxide (NDMMOH(aq)) showing improvement in terms of dissolution capacity and stability of the dissolved state (in relation to NaOH(aq)).^39^ In continuation to that work and aiming towards better understanding of the structural requirements of a solvent pertaining to enhanced cellulose dissolution, it was of high interest to investigate other morpholinium derivatives combined with not only hydroxides but also other counter ions, such as Cl^−^ and OAc^−^, both of them earlier reported as components in a number of efficient cellulose solvent.^38,40–46^ In this work, we hypothesize that the N-chain length of the morpholinium cation, as well as the nature of the counterion, critically affect solvent ability to dissolve cellulose.

Thus, we here report the development of several morpholinium derivatives with varying alkyl chain length combined with OH^−^, OAc^−^ or Cl^−^ anions and assessment of their potential towards cellulose dissolution, seeking to relate structural features to required dissolution conditions and the stability of the dissolved state. Even though use of some aprotic organic solvent could not be avoided, the synthetic procedure reflects our efforts to, as far as possible, maximize use of water-based steps, reusable ion-exchange resins and microwave-assisted reactions along with a minimized number of work-up steps.

## Experimental

### Materials

Acid washed cellulose powder from spruce with DP ∼ 350 was purchased from Fluka (product no. 22182) and dried at 60 °C in a vacuum oven for 4 h prior to use. Sulfite dissolving grade pulp from softwood with intrinsic viscosity 442 cm^3^ g^−1^ (measured by pulp dissolution in bis(ethylenediamine)copper(ii) hydroxide solution (CED) using capillary viscosimetry according to the SCAN-C 15:99 method) was provided by Domsjö. *N*-Methylmorpholine, allyl chloride bromoethane, 1-bromopropane, diethyl ether, iodomethane, 1-bromobutane and anhydrous DMSO were purchased from Sigma-Aldrich. 1-Bromoheptane were purchased from Merck and 1-bromoheptane was purchased from Alfa Aesar. Acetonitrile was provided from VWR Chemicals. Purolite SGA550OH (1.10 equiv. OH^−^ per L) as well as Amberlite-IRA410 chloride form (1.25 equiv. Cl^−^ per L) were used as the anion-exchange macroporous resin and washed with methanol before packing the column. Acetic acid and HCl were purchased from Fisher Scientific (Fisher Chemicals). All the chemicals were used as received without further purification. Silver(i) oxide was purchased from Alfa Aesar and used as received. Methanol, isopropanol, THF, ethyl acetate and acetone were provided by Friedrich-Schiller-Universität Jena.

### General procedure for the synthesis of morpholinium bromides (MorBr), the precursors for majority of synthesized salt

In a typical synthetic procedure, *N*-methylmorpholine (0.2 mol), 1-bromoalkane (0.25 mol) and acetonitrile 120 ml were added to a round bottom flask and depending on the reactivity of the corresponding 1-bromoalkene, different temperature (between 70 and 80 °C) and reaction times (usually 24 h or longer) were required as provided in detail in ESI.† The product formed was filtered off with or without addition of an anti-solvent (typically ethyl acetate), either washed or recrystallized using the desired solvent (see ESI†) and dried under vacuum at 60 °C for 24 h.

The synthesis of *N*-heptyl-*N*-methylmorpholinium bromide (HMMorBr) followed a different procedure, namely a microwave-assisted reaction due to the low reactivity of 1-bromoheptane. *N*-Methylmorpholine (0.2 mol, 22.05 ml), 1-bromoheptane (0.25 mol, 39.27 ml), and 9 ml acetonitrile were added and mixed well. The reaction was performed in capped microwave vials (3 vials were filled with maximum 20 ml of the reaction mixture) using a Biotage Initiator-8 microwave synthesizer. The temperature was set to 90 °C for 2 h with stirring rate of 780 rpm and a pre-stirring of 10 s.

### General procedure for the synthesis of morpholinium chlorides (MorCl)

A general synthesis of MorCl chlorides started from their bromide precursors. A column was packed with Purolite SGA550OH ion exchange resin with the mole ratio of 2 : 1 resin to MorBr. The desired bromide salt was dissolved in methanol to the final concentration of 0.1 M and passed through the column with the flow rate of 1 drop per second. Later, the column was washed with 400–500 ml of methanol and the solution was neutralized with 1 M HCl. Subsequent removal of the solvent using rotatory evaporation and later vacuum drying gave white hygroscopic salt. For more details on the synthesis of each salt, the reader is referred to the ESI.†

### General procedure for the synthesis of morpholinium acetate (MorOAc)

The synthesis of MorOAc started from MorBr followed by an ion-exchange of bromide to hydroxide using Purolite SGA550OH as mentioned above. After washing the column with 400–500 ml methanol, stoichiometric amount of 1 M acetic acid was added to the hydroxide solution and the mixture was stirred vigorously overnight. The excess of the solvent was removed using a rotatory evaporator and the yielded light-yellow solution was further dried in vacuum oven at 60 °C for 72 h. The water content measured by Karl-Fischer titration varied between 0.76 and 2.06 wt%.

### General procedure for the synthesis of morpholinium hydroxide (MorOH)

Aqueous solutions of MorOH were synthesized using our previously reported protocol (Naserifar *et al.*, 2021). Typically, 0.06 mol of MorBr was added to 15–20 ml distilled water in a 45 ml centrifuge vial and 0.04 mol of silver(i) oxide (9.26 g) was added to the solution. The vial was sealed tightly to avoid further contact with air and the mixture was stirred at room temperature for approx. 5 h. Colored precipitates of silver bromide were formed as soon as stirring began and by the end of the reaction, the excess grey silver(i) oxide remained unreacted. Lastly, the obtained solution was centrifuged, and the supernatant containing MorOH(aq) was isolated and kept refrigerated.

### Dissolution of cellulose powder (Fluka) and pulp (Domsjö)

#### Dissolution in MorOAc/DMSO

Due to high viscosity of MorOAc, they were shortly heated to facilitate their transfer into the dissolution set-up. MorOAc/DMSO with mole ratio of 1 : 4.6 were added to a three-neck vial equipped with a mechanical stirrer and continuous flow of nitrogen to avoid further water uptake form the air. 3 wt% cellulose powder was added, and the suspension was heated up to 120 °C under mechanical stirring. After 15 minutes the solutions became transparent.

To evaluate pulp dissolution, 1 wt% pulp was added to EMMorOAc/DMSO and stirred at 120 °C. After 20 minutes a droplet of the solution was taken for investigation using an optical microscope.

#### Dissolution in MorOH(aq)

A range of MorOH(aq) concentrations from 0.8–2.3 M was prepared by diluting the concentrated solvent with distilled water. The desired amount of cellulose powder was added to each solution to a final concentration of 3 wt% and stirred at room temperature for 5 min to yield a well-dispersed suspension. The suspension was then placed in a freezer at −25 °C for 20 min, after which it was thawed under constant stirring to remove any ice formed and thereby yield a transparent solution.

To investigate pulp dissolution, 1 wt% pulp was added to 2 M EMMorOH(aq) and stirred at room temperature. After 20 minutes a droplet of the solution was taken for investigation using an optical microscope.

### Regeneration of cellulose

Cellulose solutions were prepared according to the method described above. Then the solutions were added to large excess of ethanol and stirred for a few minutes. Then the solutions were filtered, and the precipitates were added again to ethanol and stirred properly, filtered, and washed with excess of ethanol to ensure solvent removal. Finally, the samples were air dried.

### Characterization methods

#### Characterization of the obtained MorX

##### Elemental analysis

1–2 mg of the synthesized salt was weighed using Sartorius ME36 S and the elemental analysis was performed by HEKAtech Euro EA3000 analyser.

##### Melting point

Melting point of the solids was measured using a Mettler Toledo HS1 hot stage controller equipped with HS84 DSC microscopy cell and a Zeiss microscope to monitor the melting of the crystals.

#### Nuclear magnetic resonance (NMR) spectroscopy

NMR spectra of the synthesized MorBr, MorOAc and MorCl (in D_2_O) were obtained with Bruker Avance 500 MHz spectrometers with 16 scans for ^1^H NMR (500 MHz) and up to 1024 scans for ^13^C NMR (125 MHz) at 25 °C.

NMR spectra of the synthesized NDMMI and MorOH (in DMSO-*d*_6_ or D_2_O) were obtained on a Varian (400 MHz) spectrometer equipped with One NMR probe at 25 °C.

#### Karl-Fischer titration

The amount of water in MorOAc cellulose solutions was determined by Karl-Fischer titration. Ina a typical procedure, 0.1–0.25 g of sample was accurately weighed and a coulometric Karl-Fischer-Titrator (C20S, Mettler Toledo), a cell without diaphragm and Karl-Fischer-Reagent (Aquastar®, CombiCoulomat fritless; Merck) were used for the water determination. The water content is reported in ppm.

#### Determination of MorOH(aq) concentration

Concentration of the obtained MorOH(aq) was determined using an SI analytics autotitrator. 1 ml of the MorOH(aq) was diluted with 30 ml of distilled water and titrated against 1 M HCl. The concentration was calculated using the titrant volume at the equivalence point.

#### Characterization of cellulose solutions

##### Microscopy

Dissolution of cellulose was investigated using microscopic observations (ZEISS SteREO Discovery.V12) by placing a small droplet of the solution on a slide glass that was pressed using a cover slip in a microscope using cross-polarized light equipped with a camera at ambient temperature. Dissolution was confirmed when no particles could be detected, whereas the solubility limit was reached as soon as a crystal could be observed.

#### Nuclear magnetic resonance (NMR) spectroscopy to assess cellulose solution stability

**Fig. 1:**
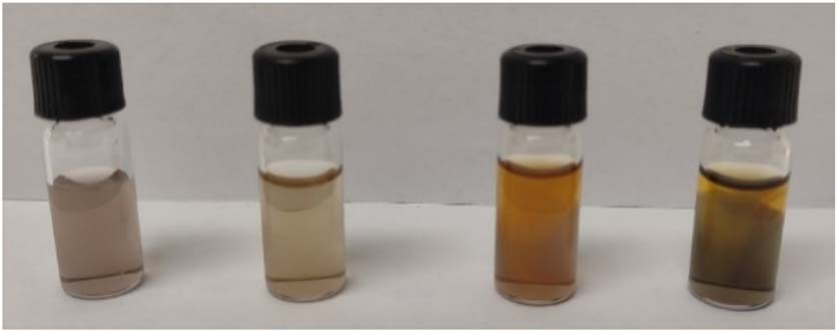
3 wt% cellulose dissolved in MPMorOH(aq) from left to right 1 M, 1.3 M, 1.8 M, 2.1 M after storage in the fidge overnight.

To assess the stability of cellulose solutions in MorOH(aq) as they were prone to color change over time (Fig. 1), freshly dissolved cellulose (4 wt%) in 1.3 M BMMorOH(aq) and 1.3 M MPMorOH(aq) were subjected to ^13^C NMR measurements and spectra were collected every 1 h over 22 h on the same sample.

#### Rheological investigations of the cellulose solutions

Flow sweep measurements of cellulose solutions in different solvents at 0, 25, 50 and 55 °C at shear rates of 1–500 s^−1^ were measured using a TA Discovery Hybrid Rheometer (HR-3), with a sandblasted 40 mm plate–plate geometry with a gap of 500 μm and the temperature was controlled with a Peltier plate with circulating cooling liquid. Samples were measured directly after dissolution, a water-filled solvent trap was used to avoid solvent evaporation while running rheology measurements and the rheometer was brought to the desired temperature without pre-shearing. In addition, increasing temperatures was conducted in series on the same sample.

When running measurements using morpholinium acetates silicon oil was applied around the border of the geometry after applying the selected gap as well as the solvent trap to avoid the moisture uptake.

#### Characterization of regenerated cellulose

##### Size exclusion chromatography (SEC)

A chromatography system from Polymer Laboratories, comprised of a PL-GPC 220 with an RI detector, was employed for SEC. It included a set of guard column mixed-A 20 μm (7.5 × 50 mm) and 2 mixed-A 20 μm (300 × 7.5 mm), also from Polymer Laboratories, connected in series. The mobile phase contained 0.5 w/v% LiCl/DMAc with flow rate of 1 ml min^−1^ operating at 70 °C. Pullulan polysaccharides obtained from Polymer Lab with molecular masses of 708 000, 344 000, 200 000, 107 000, 47 100, 21 100, 9600 and 6100 Da were used as calibration standards.

Prior to analysis, 25 mg of the sample was solvent-exchanged 3 times with 5 ml of methanol for 30 min, followed by a further 3 time solvent exchange step using DMAc for 30 min. The excess of DMAc was then removed and 5 ml of 8% (w/v) LiCl/DMAc was added and left overnight at ambient temperature with mild magnetic stirring. This sample was thereafter diluted with 20 ml of DMAc.

#### Solid state NMR

Regenerated cellulose was subjected to solid-state NMR experiments carried out on a Bruker Avance III 500 MHz spectrometer equipped with a 4 mm HX CP MAS probe. Experiments were recorded at a magic angle spinning (MAS) rate of 10 kHz and the temperature was set to 298 K. ^1^H decoupling with a ‘‘spinal64’’ decoupling scheme at 67 kHz was applied during the acquisition. The cross-polarization (CP) contact time was set to 1.5 ms and the repetition time to 2 s. CP spectra for comparison were recorded with 4000 signal accumulations. The crystallinity was estimated according to Sparrman *et al.*^47^ using 128 signal accumulations using the integral of C1.

## Result and discussion

**Scheme 1:**
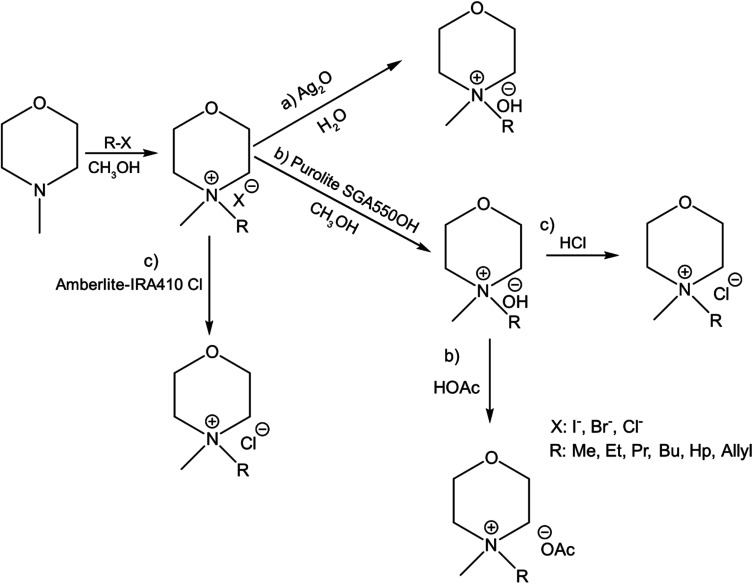
Overview of the principal procedures used for the synthesis of morpholinium salts: the pathway (a) leading to MorOH(aq) and (b) MorOAc and (c) showing routes used to prepare the chlorides. Details of the procedures are found in the Experimental section as well as ESI.†

In continuation to our previous work on NDMMOH(aq) as a cellulose solvent, a series of morpholinium cations with different alkyl chains were synthesized and combined with OH^−^, Cl^−^ and OAc^−^ as counterions following the general procedure shown in Scheme 1 and investigated for their ability to dissolve cellulose.

### The impact of alkyl chain length on cellulose dissolution in MorOH(aq)

The obtained hydroxides were investigated for the ability of their aqueous solutions to dissolve cellulose. More specifically, the dissolution of 3 wt% cellulose (the amount commonly dissolved by the previously studied aqueous hydroxides) by the aqueous hydroxides was assessed using freeze–thaw method or stirring the solution at room temperature followed by polarized light microscopy studies. Freeze–thaw method is commonly employed to enable cellulose dissolution in aqueous hydroxides, restricted to low temperatures and possibly dependent on the impact of freezing and thawing on cellulose morphology. All the synthesized compounds dissolved cellulose in a concentration range of 1–2.3 M by freeze–thaw method (optical microscopy of the undissolved as well dissolved cellulose can be found in ESI†). Interestingly, the dissolution window of HMMorOH(aq), the hydroxide with the longest alkyl chain investigated, extended to slightly lower concentration of the hydroxide to 0.8 M. Even more interestingly, the hydroxides bearing the alkyl chain longer than ethyl could dissolve cellulose at room temperature too (Table 2, details on the abbreviations are listed in Table 1) and as the chain length increased, lower solvent concentration was required for room temperature dissolution.

**Table 1:** List of abbreviations used to name the compounds

Abbreviation	Compound name
NDMMOH	*N*,*N*-Dimethylmorpholinium hydroxide
EMMorOH	*N*-Ethyl-*N*-methylmorpholinium hydroxide
MPMorOH	*N*-Methyl-*N*-propylmorpholinium hydroxide
BMMorOH	*N*-Butyl-*N*-methylmorpholinium hydroxide
HMMorOH	*N*-Heptyl-*N*-methylmorpholinium hydroxide
EMMorOAc	*N*-Ethyl-*N*-methylmorpholinium acetate
MPMorOAc	*N*-Methyl-*N*-propylmorpholinium acetate
BMMorOAc	*N*-Butyl-*N*-methylmorpholinium acetate
HMMorOAc	*N*-Heptyl-*N*-methylmorpholinium acetate
NDMMCl	*N*,*N*-Dimethylmorpholinium chloride
EMMorCl	*N*-Ethyl-*N*-methylmorpholinium chloride
MPMorCl	*N*-Methyl-*N*-propylmorpholinium chloride
AMMorCl	*N*-Allyl-*N*-methylmorpholinium chloride

**Table 2:** Dissolution of cellulose in MorOH(aq)

Solvent	Freeze–thaw dissolution	Solvent concentration limit for freeze–thaw dissolution	r.t. dissolution	Solvent concentration required for r.t. dissolution
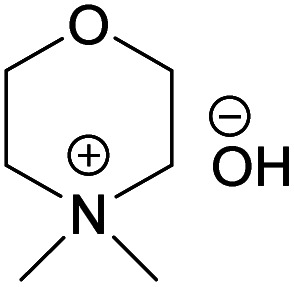 (NDMMOH)	✓	1–2 M	✗	—
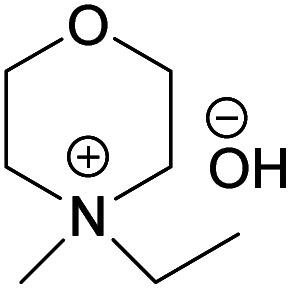 (EMMorOH)	✓	1–2.3 M	✗	—
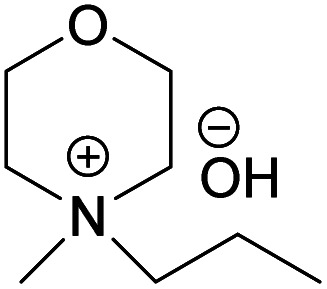 (MPMorOH)	✓	1–2.3 M	✓	2.1 M
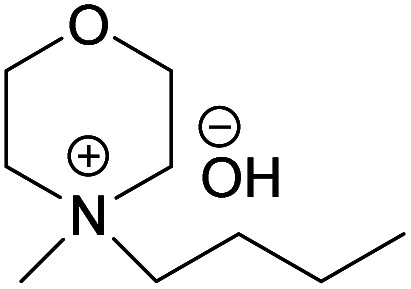 (BMMorOH)	✓	1–2.3 M	✓	2 M
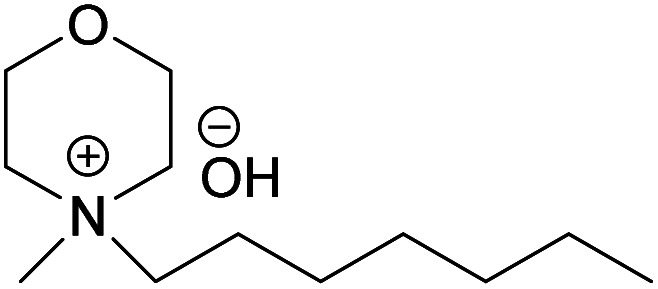 (HMMorOH)	✓	0.8–2.3 M	✓	1.9 M

This finding points out towards a beneficial impact of increased chain length of the morpholinium cation on cellulose dissolution. Indeed, enhanced cellulose dissolution in aqueous solvents through increase in cationic hydrophobicity has been previously reported.^48^ Low temperature requirement for cellulose dissolution in aqueous hydroxides is not yet fully understood but is probably associated with cellulose amphiphilicity and the requirement to stabilize both hydrophilic and hydrophobic cellulose regions through cellulose–solvent interactions. For instance, according to Lindman *et al.*, glucose ring conformation at low temperature would reduce tendency for cellulose–cellulose interactions through hydrophobic pairing by minimizing the exposure of its hydrophobic regions and thereby promote dissolution.^49^ In light of that, one explanation of the obtained results in this work can be that increase in alkyl chain length provides sufficiently effective interaction between the morpholinium cations and cellulose hydrophobic regions even at room temperature and, consequently, also prevents cellulose chain reassociation through hydrophobic pairing.

Interestingly, in spite of these differences, investigations of maximum solubility of cellulose in 1.3 M MorOH(aq), showed that in all of the solvents maximum 7 wt% of cellulose could be dissolved similar to NDMMOH(aq) reported in the previous study.^39^

### Are cellulose solutions in MorOH(aq) chemically stable over time?

**Fig. 2:**
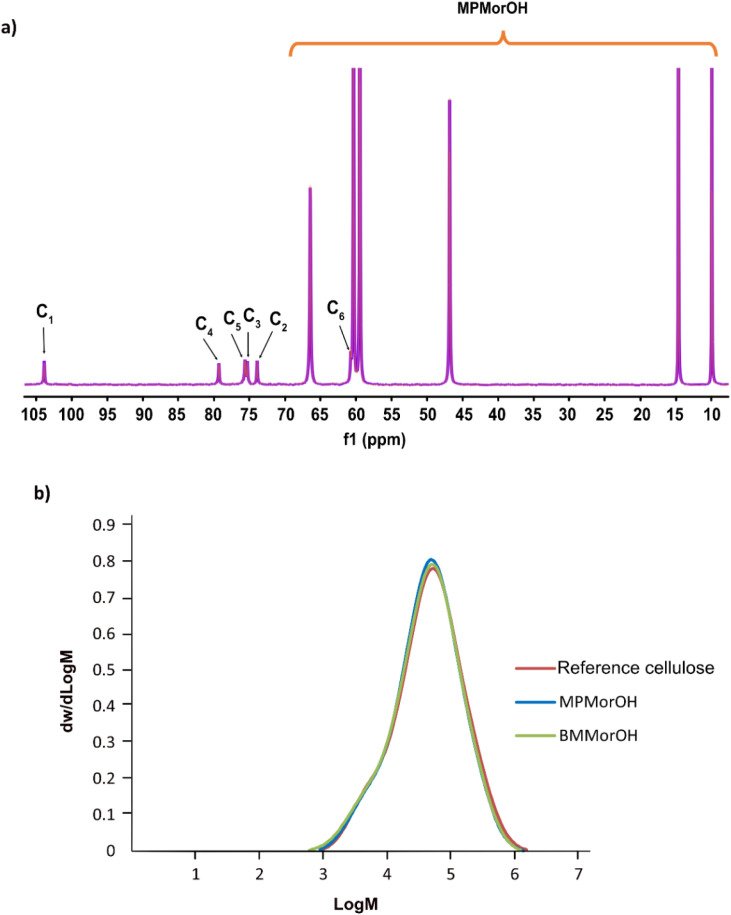
(a) ^13^C NMR spectra of 4 wt% freshly dissolved cellulose in 1.3 M MPMorOH(aq) (orange) and an aged solution for 22 h post dissolution (purple) (due to perfect overlap of both spectra the orange spectrum cannot be seen). (b) Molecular weight distribution (determined by SEC in DMAc/LiCl) for cellulose as the reference (red) and cellulose dissolved and precipitated after 24 h storage from MPMorOH(aq) (blue) and BMMorOH(aq) (green).

As chemical instability in cellulose hydroxide solutions is always a possibility considering rather extreme dissolution conditions, it was of high interest to assess the stability of these cellulose solutions, especially that while dissolving cellulose, an immediate appearance of a yellow color that darkened quickly at higher solvent concentrations was observed (Fig. 1). The light yellow color has been observed before in NDMMOH(aq) but not as dark as with the synthesized MorOH(aq) in this study. However, monitoring the 4 wt% cellulose solutions in 1.3 M BMMorOH(aq) and MPMorOH(aq) over 22 h by ^13^C NMR showed no detectable indications of degradation (Fig. 2a and S18 in ESI†).

Similarly, molecular weight distribution measurements in DMAc/LiCl on regenerated cellulose (3 wt%) from 1.3 M MPMorOH(aq) and 1.3 M BMMorOH(aq) after 24 h storage at room temperature as well as untreated cellulose as a reference did not show any significant degradation of cellulose in any of the solvents (Fig. 2b). A very minor decrease in *M*_w_ observed can be probably ascribed to a common beta-elimination starting from the reducing-ends of cellulose when it is well dissolved and highly accessible under alkaline conditions. Thus, cellulose solutions at room temperature were in principle chemically stable; the observed color possibly being a result of reducing end reactions generating low amounts of strong chromophores. Detailed SEC analysis data can be found in ESI.†

### Dissolving cellulose in MorOAc and MorCl

All the synthesized MorOAc (Table 3, details on the abbreviations are listed in Table 1), were liquid at room temperature, highly viscous and contained some water though they were vacuum dried for three days or longer. Traces of water in ionic liquids are almost unavoidable.^50,51^ Interestingly, as the alkyl chain length increased the water content decreased except for MPMorOAc. Similar effect of alkyl chain length was reported for other types of ionic liquids: the increase in alkyl chain length and thus increase in hydrophobicity resulted in lower water uptake.^52–54^

**Table 3:** Cellulose dissolution capacity in the studied MorOAc/DMSO along with the content of the hard-to-remove water in MorOAC

Solvent	Maximum dissolved cellulose (wt%)	Water content in MorOAc (wt%)
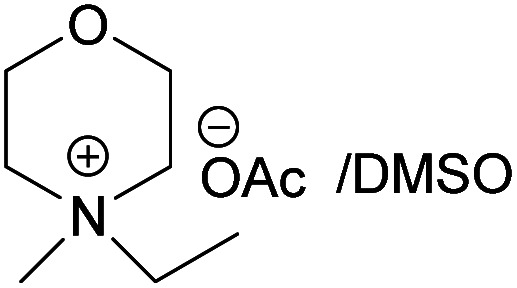 (EMMorOAc)	14	1.28
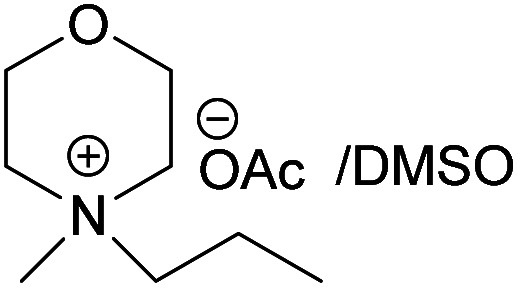 (MPMorOAc)	3	2.06
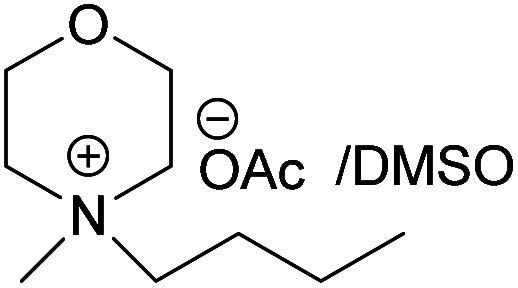 (BMMorOAc)	10	0.86
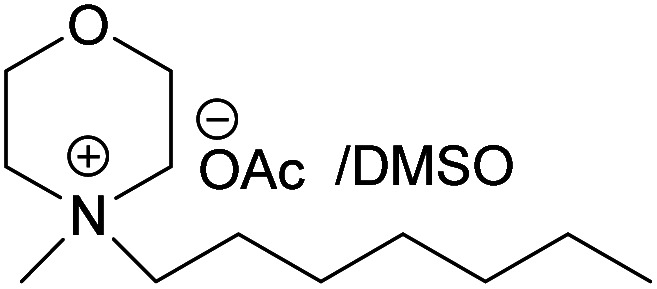 (HMMorOAc)	10	0.76

**Fig. 3:**
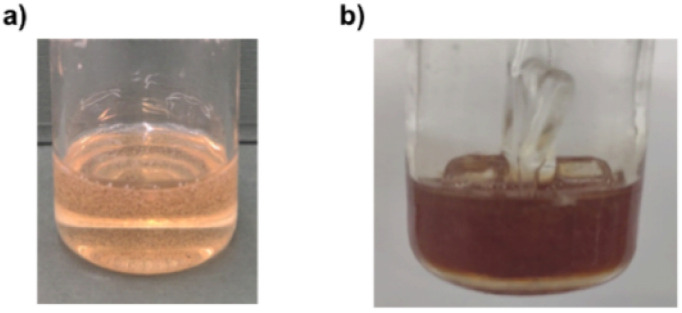
(a) 3 wt% (b) 8 wt% cellulose dissolved in HMMorOAc/DMSO.

Surprisingly, none of the MorOAc were able to dissolve cellulose over a large temperature range, even at temperatures as high as 120 °C where they showed ionic liquid behavior. Even though MorOAc exhibit good mobility at elevated temperature (behaving as ionic liquids), their ions seem not to be capable of penetrating and accessing cellulose structure, most probably due to large ion sizes (where hydration with water is probably one of the contributing factors). However, in combination with DMSO, a strong swelling agent, with molar ratio of 1 : 4.6 MorOAc/DMSO dissolution was achieved at 120 °C within 15 minutes (optical microscopy of the undissolved as well as the dissolved cellulose can be found in ESI†). Addition of DMSO probably promotes dissolution by swelling cellulose,^55^ making it accessible also for larger ions, as well as enhancing the mass transport by decreasing the viscosity of MorOAc.^56,57^ In addition, it has been shown that solvation of IL ions by DMSO increases conductivity and ion mobility resulting in dissociation of ions.^58^ The DMSO solutions of acetates were yellow prior to cellulose addition and as the concentration of cellulose in the solution increased, an increase in viscosity was observed while the solution turned browner as represented in Fig. 3.

Similarly, the high temperature required for cellulose dissolution in ILs has been previously reported to promote dissociation of the ion pairs promoting, in turn, the interaction between the anion and cellulose hydroxyl groups while the cation possibly associates with cellulose hydroxyl oxygens affecting hydrogen bonding ability of cellulose.^59^ Among the investigated solvents EMMorOAc showed the highest dissolution capacity of 14 wt% cellulose while having 1.28 wt% water content. Notably, MPMorOAc, containing the highest amount of hard-to-remove water (2.06%, Table 3), showed the lowest cellulose dissolution of 3 wt%. Different ionic liquids have different water limit to dissolve cellulose.^60–62^ The higher water content in MPMorOAc might be the reason for its lower dissolution capability. It is worth mentioning that the efforts to lower the water content from MPMorOAc by longer vacuum drying, failed possibly due to water being bound to the IL. However, the water content does not seem to be the only determinant for the dissolution ability, as both BMMorOAc and HMMorOAc showed lower dissolution capacity (10 wt%) than EMMorOAc despite containing less water. Surprisingly, in contrast to the hydroxides, the dissolution ability of acetates does not seem to be improved by increasing the length of the alkyl chain. Studies on imidazolium ILs in which the increase in alkyl chain length was shown to lead to decreased cellulose dissolution,^62–64^ interpret this observation as a result of a steric hindrance for the anion interaction with cellulose; thus, the shorter alkyl chain of the cation would allow for a stronger interaction between the IL and cellulose. In similar studies, it has been argued that the smaller cation would permit more anions to enrich in proximity of the polymer promoting dissolution.^62^ However, in case of these particular morpholinium-based solvents further detailed investigation is needed, taking also into consideration the amount of water bound by the IL.

**Fig. 4:**
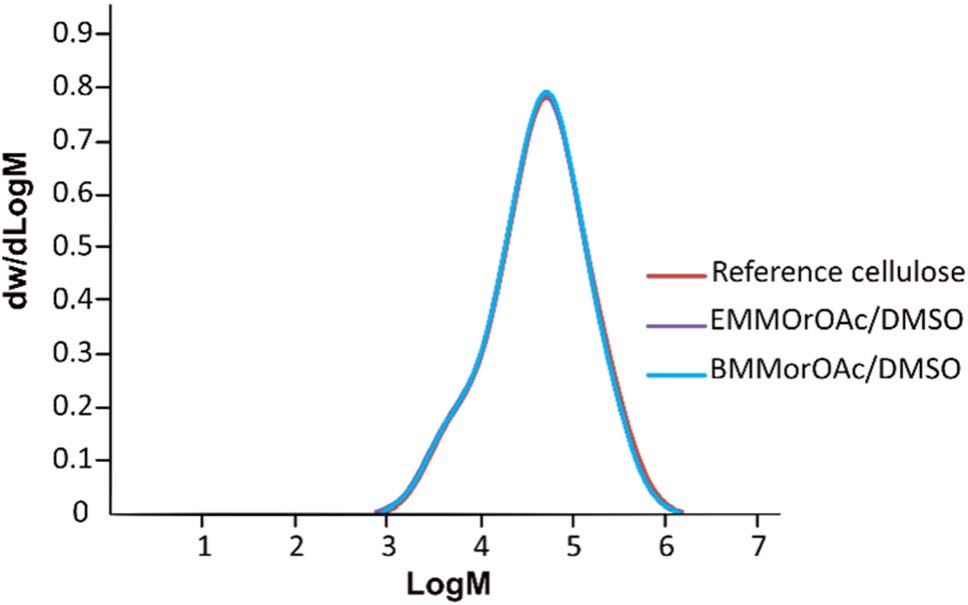
Molecular weight distribution (determined by SEC in DMAc/LiCl) for cellulose as the reference (red) and cellulose dissolved and precipitated after 24 h storage from EMMorOAc/DMSO (purple) and BMMorOAc/DMSO (blue) (1 : 4.6 mole ratio).

Similar to MorOH(aq), SEC results on cellulose precipitated from EMMorOAc/DMSO and BMMorOAc/DMSO (Fig. 4) revealed a chemical integrity of cellulose as no significant deviations in molecular weight distribution could be observed upon storage at room temperature for 24 h. Detailed SEC analysis data summarized in Table S1 can be found in ESI.†

When it comes to the prepared chloride salts (Table 4, details on the abbreviations are listed in Table 1, details on the synthesis procedure are available in ESI†), all compounds were hygroscopic solids and had high melting points, in general over 180 °C. Both water and polar aprotic cosolvents, namely DMSO, DMAc and acetone, were combined with the synthesized salts in an attempt to dissolve cellulose. Yet, none of these combinations proved successful, not even at higher temperatures. Insolubility of cellulose in water solutions of the chloride salts is not a surprise, as water systems, unless containing components capable of deprotonating cellulose or making a strong complex with its hydroxyls, are in general non-solvents. The reason being, likely, the extensive hydration of the ions excluding interactions with cellulose hydroxyls, combined with a poor swelling and inaccessibility of cellulose in the absence of charging species (or species with the ability to make a complex) that would trigger diffusion of water into the cellulose structure. Even in the water-poor systems, such as ionic liquids (*e.g.* BMIMCl) and salts in aprotic organic solvent (*e.g.* LiCl/DMAc, TBAF/DMSO) the presence of water is well-known to hamper the dissolution.^62,65^ On the other hand, explanation for the insolubility in the polar aprotic solvents might be found in the inability of these solvents to dissociate the ion pairs (probably due to the strong interaction of selected morpholinium cations with chloride). Previous results on cellulose dissolution in quaternary ammonium fluoride (QAFs) showed a correlation between the dissolution of these salts in an aprotic polar solvent such as DMSO and their ability to dissolve cellulose: their dissociation in DMSO was pointed out as requirement for an effective interaction with cellulose anhydroglucose units.^66^

**Table 4:** Synthesized MorCl

Compound	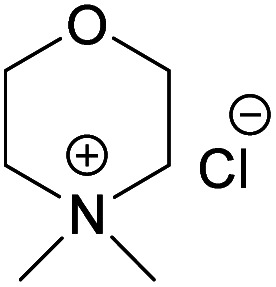 (NDMMCl)	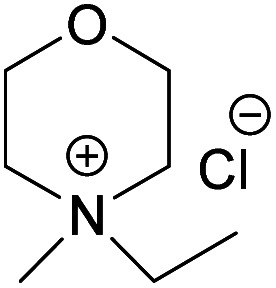 (EMMorCl)	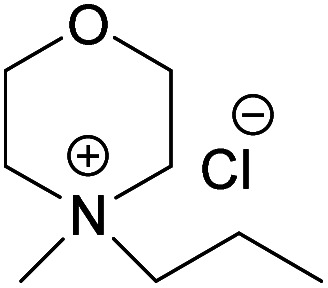 (MPMorCl)	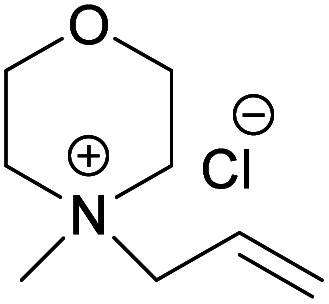 (AMMorCl)
Melting point (°C)	>300	217	244.5	182.5

### Flow properties of the obtained cellulose solutions at different temperatures

**Fig. 5:**
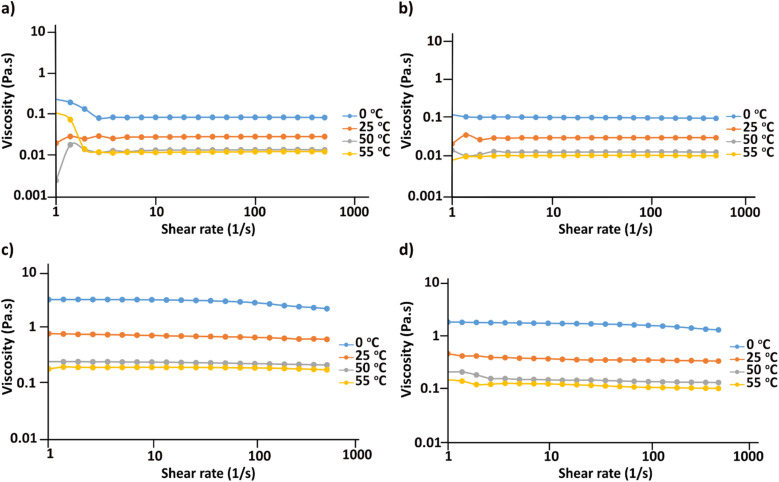
Flow sweep curves of 3 wt% cellulose dissolved in (a) 1.3 M MPMorOH (b) 1.3 M BMMorOH (c) EMMorOAc/DMSO (d) MPMorOAc/DMSO.

In order to obtain information on the stability of the cellulose solutions in the obtained hydroxides and acetates at different temperatures, a series of flow sweep measurements were run applying shear rate of 1–500 s^−1^ at 0, 25, 50 and 55 °C. As shown in Fig. 5 all the solutions show Newtonian behavior during the measurement interval and at the applied temperatures and upon increase in temperature the viscosity of all the solutions decreases. No noticeable aggregates could be detected which further confirms the solution stability at different temperatures while applying low to high shear rates. Previous studies on cellulose solutions in NaOH(aq), tetramethylammonium hydroxide (TMAH), benzyltrimethylammonium hydroxide (Triton B)^67^ showed that in NaOH cellulose always shows shear thinning behavior indicative of cellulose aggregates existence within the solution. However, when mixed with either TMAH or Triton B, it showed non-Newtonian behavior at high temperatures. In addition, cellulose in TMAH(aq) showed possible aggregation at low temperatures. Therefore, the obtained results in this study prove enhanced temperature stability of the dissolved state in both MorOH(aq) as well as MorOAc/DMSO. Not surprisingly, the MorOH(aq) solutions have lower viscosity than MorOAc/DMSO that also show higher viscosity dependency on temperature. It is worth mentioning that few distorted points are observed in Fig. 5a at the beginning of the measurement which are sourced by the rheometer error.

### Assessment of cellulose crystallinity after regeneration from MorOH(aq) *vs.* MorOAc/DMSO

**Fig. 6:**
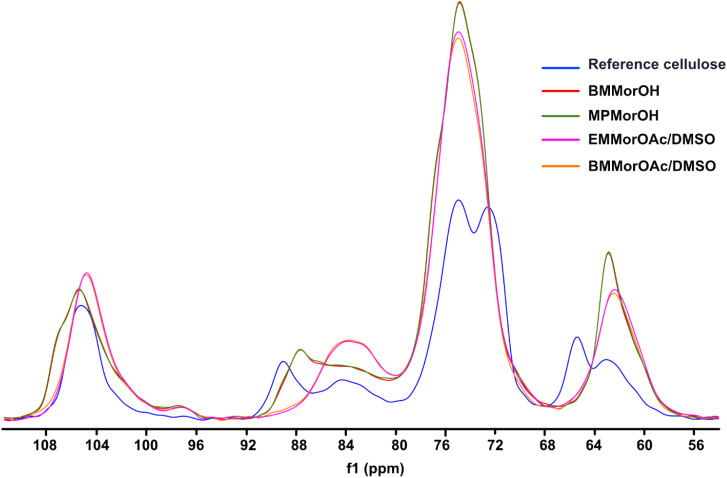
CP/MAS ^13^C NMR of reference cellulose and cellulose regenerated in different solvents.

According to Fig. 6, cellulose regenerated from hydroxide solutions (BMMorOH(aq) and MPMorOH(aq)) show mutually identical spectra. Likewise, samples regenerated from the two acetates (BMMorOAc/DMSO and EMMorOAc/DMSO) show completely overlapping spectra. However, the two sets of spectra – those of cellulose regenerated from MorOH(aq) and from MorOAc/DMSO, respectively – look different, indicating different regeneration from these two solvent groups. The reference cellulose spectrum (blue) shows the C1 peak at 105.15 ppm, C4 peak 89.03 ppm (crystalline) and 84.04 ppm (amorphous), C2, C3 and C5 peaks at 74.97 and 72.16 ppm, C6 peak at 65.45 ppm (crystalline) and 62.48 ppm (amorphous). Upon regeneration, all the samples showed an overlapping signal of C2, C3 and C5 at around 75 ppm. The shift of the peak at 65.45 to 62.17 ppm in MorOAc/DMSO and 62.74 ppm in MorOH(aq) is suggesting that the “*t*–*g*” conformation of the C6–OH group for the crystalline parts of cellulose I had changed into a “*g*–*t*” conformation of cellulose II.^68,69^ According to previous reports, formation of cellulose II is indicated when two characteristic peaks at *ca.* 107 and 87.5 ppm appear.^70–74^ Indeed, for the samples regenerated from MorOH(aq) a shoulder at 107.1 and a new peak at 87.7 ppm appear besides the increase in the peak intensity at 84 and 62.74 ppm corresponding to amorphous cellulose. However, cellulose regenerated from MorOAc/DMSO does not show the appearance of the two aforementioned cellulose II peaks, yet an increase in intensity of the peak at 84 and 62.1 ppm indicative of amorphous cellulose. Enhanced formation of crystalline regions during coagulation in the presence of water has been previously reported and attributed to a faster diffusion of solvent in the aqueous media promoting formation of crystalline aggregates, along with enchanced relaxation of cellulose chains during drying in the presence of water.^75,76^ The main difference between the coagulation from hydroxides and acetates when ethanol is used as coagulant in this study is, thus, probably related to their water content leading to faster diffusion of hydroxides allowing for crystalline material (cellulose II) to be obtained.

Degree of crystallinity was calculated using solid state NMR according to the previously reported method.^47^ This method estimates the crystallinity using the integrals obtained from two different pulse sequences optimized for the difference in ^13^C *T*_1_ relaxation times for amorphous and crystalline cellulose. Here, the C1 integrals were used since the C4 and C2,3,5 peaks were overlapping. The estimated crystallinity for the reference cellulose was 0.44 which decreased upon dissolution in MorOH(aq) and MorOAc/DMSO. The estimated values were as follows: 0.36 for regenerated cellulose from BMMorOH(aq) and MPMorOH(aq), 0.29 and 0.23 for regenerated cellulose from BMMorOAc/DMSO and EMMorOAc/DMSO respectively.

### Dissolution of pulp in MorOH(aq) *vs.* MorOAc/DMSO

With the ability of the MorOH and MorOAc/DMSO to dissolve cellulose and generate stable solutions established, it was highly interesting to attempt dissolving cellulose pulps.

**Fig. 7:**
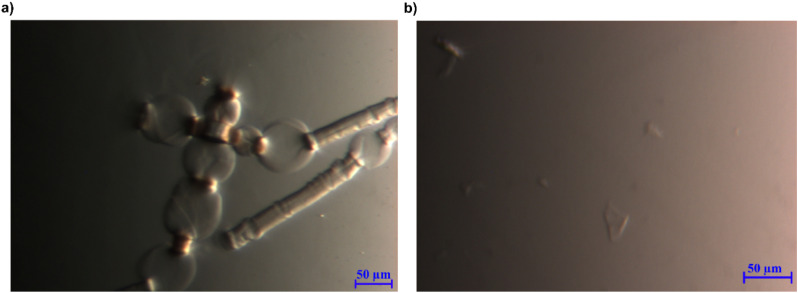
(a) 1 wt% pulp in 2 M EMMorOH(aq) at r.t. (b) 1 wt% pulp in EMMorOAc/DMSO at 120 °C.

When 1 wt% pulp was added to EMMorOH(aq), the fibres were swollen and showed ballooning effect (Fig. 7a) which is a step preceding a complete dissolution. Indeed, after keeping the same sample in the freezer at 25 °C for 20 minutes and thawing it, no undissolved cellulose was detected. EMMorOAc/DMSO also demonstrated ability to dissolve majority of pulp (1 wt%). However, optical microscopy results showed a few undissolved particles (Fig. 7).

## Discussion

In summary, varying the alkyl chain length and the counterion, provided some valuable insights in structural features governing ability of morpholinium derivatives to dissolve cellulose. While ability to interact with cellulose (through H-bonding, dispersion, or electrostatic interactions) is important, the accessibility of the structure seems to be the prerequisite allowing for interactions and thus, being completely decisive. Only structures capable of swelling cellulose were successful solvents: alkaline solutions of morpholinium hydroxides and DMSO-solutions of morpholinium acetates. Insolubility of chlorides in strong swelling agents (DMSO, DMAc) also prevented interaction with cellulose. Once access to the cellulose structure and thus cellulose–solvent interactions were enabled through swelling, length of the alkyl chain became important, but showed, interestingly enough, different effects in water and DMSO systems. In water, where stabilizing hydrophobic regions of cellulose is important, the increased chain length provided both a somewhat expanded concentration range for dissolution and a room temperature solubility (for alkyl chains longer than ethyl). In the DMSO/MorOAc systems, on the other hand, the increased length of the alkyl chain (butyl and heptyl) led to a decreased amount of bound water, but possibly also to a sterical hindrance for cellulose–anion interactions resulting in varied dissolution effects. In general, though, MorOAc/DMSO showed higher dissolution capacity compared to the MorOH(aq) along with a lower crystallinity upon coagulation with ethanol. While the lower crystallinity of cellulose coagulated under principally water-free conditions has been observed before, the question of the factors limiting dissolution capacity in aqueous cellulose solutions calls for further investigation.

## Conclusion

A series of morpholinium cations were combined with OH^−^, OAc^−^ and Cl^−^ to assess their ability towards cellulose dissolution. MorOH(aq) were able to dissolve cellulose by freeze–thaw method while chain lengths longer than ethyl enabled room-temperature dissolution. As a result of alkyl chain length increase the solvent concentration required for room-temperature dissolution decreased. It is assumed that the increase in hydrophobicity of the cation enhances hydrophobic interaction between the cation and cellulose thus preventing cellulose chain reassociation. Morpholinium acetates solely did not dissolve cellulose despite increase in the temperature but required combination with a strong swelling agent DMSO to dissolve cellulose at 120 °C. Increase in alkyl chain length of acetates affected both their tendency to retain water and possibly also ability of the acetate anion to interact with cellulose through an increasing steric effect. In spite of decrease in water content a lower dissolution ability was observed when alkyl chain length increased. The highest amount of dissolved cellulose was 14 wt% reported for EMMorOAc/DMSO while in MorOH maximum 7 wt% cellulose could be dissolved. Yet, all the prepared morpholinium chlorides had high melting points and were not soluble in common organic solvents such as acetone, DMSO or DMAc, failed in dissolving cellulose. This work has expanded the knowledge on the design of aqueous and organic morpholinium-based solvents while the easy synthesis, increased dissolution capacity and possibility of cellulose dissolution in MorOH(aq) make them appropriate for further research and application in cellulose field.

## Author contributions

Shirin Naserifar: conceptualization, formal analysis, investigation, writing – original draft, visualization. Andreas Koschella: conceptualization, supervision, writing – review & editing. Thomas Heinze: conceptualization, writing – review & editing. Diana Bernin: methodology, investigation, writing – review & editing. Merima Hasani: conceptualization, supervision, project administration, funding acquisition, writing – review & editing.

## Conflicts of interest

There are no conflicts to declare.

## Supplementary Material

RA-013-D3RA03370H-s001
