# Correction: Investigation of cellulose dissolution in morpholinium-based solvents: impact of solvent structural features on cellulose dissolution

**DOI:** 10.1039/d3ra90120c

**Published:** 2023-12-01

**Authors:** Shirin Naserifar, Andreas Koschella, Thomas Heinze, Diana Bernin, Merima Hasani

**Affiliations:** a Department of Chemistry and Chemical Engineering, Chalmers University of Technology 412 96 Gothenburg Sweden shirinn@chalmers.se +46317722999; b Wallenberg Wood Science Center, Chalmers University of Technology 412 96 Gothenburg Sweden; c Center of Excellence for Polysaccharide Research, Institute of Organic Chemistry and Macromolecular Chemistry, Friedrich Schiller University of Jena Humboldtstraße 10 07743 Jena Germany

## Abstract

Correction for ‘Investigation of cellulose dissolution in morpholinium-based solvents: impact of solvent structural features on cellulose dissolution’ by Shirin Naserifar *et al.*, *RSC Adv.*, 2023, **13**, 18639–18650, https://doi.org/10.1039/D3RA03370H.

Following publication of the original manuscript, an additional note is required to improve the reader's understanding in this article and to remove any confusion around the discussion of crystallinity in the section “Assessment of cellulose crystallinity after regeneration from MorOH(aq) *vs.* MorOAc/DMSO”. A Correction is required to clarify the discussion in this section.

In the paragraph that begins with “Degree of crystallinity…” on page 18647, the authors wish to add a note to mention that the technique used is based on some assumptions. In this particular case it is assumed that the *T*_1_ relaxation time for amorphous cellulose is one or two magnitudes shorter than for crystalline cellulose, yet residual solvent and coagulant might affect this assumption. This method was applied as a means of comparison between different coagulated cellulose samples and the estimated values should be considered as trends in crystallinity, which were used to confirm the qualitative analysis reported in the previous paragraph when interpreting the ^13^C CP/MAS spectra.

This does not affect the conclusions or any other part of the paper.

The Royal Society of Chemistry apologises for these errors and any consequent inconvenience to authors and readers.

## Supplementary Material

