# Supplementary material for: Investigation of cellulose dissolution in morpholinium-based solvents: impact of solvent structural features on cellulose dissolution
Source: RSC Adv. 2023 Jun 20;13(27):18639–50. doi: 10.1039/d3ra03370h (PMC10280132; doi:10.1039/d3ra03370h)
Supplement: RA-013-D3RA03370H-s001 [file RA-013-D3RA03370H-s001.pdf]

Electronic Supplementary Material (ESI) for RSC Advances.
This journal is © The Royal Society of Chemistry 2023

Supporting information

Investigation of cellulose dissolution in morpholinium-based solvents: Impact of solvent structural features on cellulose dissolution

Shirin Naserifar[*,a,b], Andreas Koschella[c], Thomas Heinze[c], Diana Bernin [a] and Merima Hasani[a,b]

[a]Department of Chemistry and Chemical Engineering, Chalmers University of Technology, 412 96 Gothenburg, Sweden.

[b]Wallenberg Wood Science Center, Chalmers University of technology, 412 96 Gothenburg, Sweden.

[c]Center of Excellence for Polysaccharide Research, Institute of Organic Chemistry and Macromolecular Chemistry, Friedrich Schiller University of Jena, Humboldtstraße 10, 07743 Jena, Germany

Synthesis of 4-ethyl-4-methylmorpholinium bromide (EMMorBr)

In a typical run 0.2 mol *N*-methylmorpholine (22.05 ml), 0.25 mol bromoethane (18.65 ml) and 120 ml acetonitrile were added to a round bottom flask and the reaction was left to stir at room temperature overnight. White crystals appear and to complete the reaction the solution was refluxed at 70 °C for 6.5 h. After completion of the reaction, the solution was kept in a freezer at -18 °C for 1 h to allow crystallization of the product. Finally, the precipitates were filtered, washed three times with sufficient amount of ethyl acetate and dried in a vacuum oven at 50 °C for 24 h. The dry product was stored in a desiccator to avoid any moisture uptake from atmosphere.

White hygroscopic solid. Elemental analysis for $C_7H_{16}NOBr$ calc. (%): C 40.01, H 7.68, N 6.67. Found: C 39.80, H 7.85, N 6.54.

Synthesis of 4-methyl-4-propylmorpholinium bromide (MPMorBr)

In a typical run 0.2 mol *N*-methylmorpholine (22.05 ml), 0.25 mol bromopropane (22.77 ml) and 120 ml acetonitrile were added to a round bottom flask and the reaction was carried out at 70 °C for 24 h under reflux. After completion of the reaction, ethyl acetate was added to the solution to allow the product diffusion out of the solution. Finally, the precipitates were filtered, washed three times with sufficient amount of ethyl acetate and dried in a vacuum oven at 50 °C for 24 h. The dry product was stored in a desiccator to avoid any moisture uptake from atmosphere.

Light orange solid. Elemental analysis for $C_8H_{18}NOBr$ calc. (%): C 42.87, H 8.09, N 6.25. Found: C 42.86, H 8.06, N 6.26.

Synthesis of 4-butyl-4-methylmorpholinium bromide (BMMorBr)

In a typical run 0.2 mol *N*-methylmorpholine (22.05 ml), 0.25 mol bromobutane (26.9 ml) and 120 ml acetonitrile were added to a round bottom flask and the reaction was carried out at 70 °C for 24

h under reflux. By the end of the reaction some precipitates were formed however to allow full crystallization of the product, the solution was kept in a freezer at -18 °C for 1 h. After that addition of ethyl acetate would help diffuse more products out. Finally, the precipitates were filtered, washed three times with sufficient amount of ethyl acetate and dried in a vacuum oven at 50 °C for 24 h. The dry product was stored in a desiccator to avoid any moisture uptake from atmosphere.

Light pink solid. Elemental analysis for $C_7H_{16}NOBr$ calc. (%): C 45.39, H 8.46, N 5.88. Found: C 45.56, H 8.39, N 5.91.

Synthesis of 4-hyptyl-4-methylmorpholinium bromide (HMMorBr)

The reactivity of bromoalkanes decreases as the alkyl chain increases thus longer reaction times and higher temperature is required to obtain the desired products. Here, microwave irradiation was used to help accelerate the reaction. In a typical run 0.2 mol *N*-methylmorpholine (22.05 ml), 0.25 mol 1-bromoheptan (39.27 ml) and 15 ml acetonitrile were added to a beaker and stirred for a few minutes. Then the solution was poured into four vials up to maximum 20 ml. The microwave oven was set to 80 °C for 2 h and the vials were put in a queue to run consecutively. After 2 h dark brown solution was obtained and in which crystals appeared after a few minutes. Finally, ethyl acetate was added to each vial to allow full crystallization of the product. The precipitates were filtered and recrystallizes using acetone/ethyl acetate. washed three times with sufficient amount of ethyl acetate and dried in a vacuum oven at 50 °C for 24 h. The dry product was stored in a desiccator to avoid any moisture uptake from atmosphere.

White solid 89.3% Elemental analysis for $C_{12}H_{25}NOBr$ calc. (%): C 51.43, H 9.35, N 5. Found: C 50.69, H 9.42, N 4.96.

Synthesis of 4-allyl-4-methylmorpholinium chloride (AMMorCl)

0.2 mol *N*-methyl morpholine (22.05 ml) was added to 50 ml acetonitrile and then 0.3 mol allylchloride (24.44 ml) was added to the solution. The solution was stirred at room temperature and solid precipitates appeared quickly. After 4 h the precipitates were collected, washed with diethyl ether and oven dried.

White hygroscopic solid. Elemental analysis for $C_8H_{16}NOCl$ calc. (%): C 54.08, H 9.08, N 7.88. Found: C 53.95, H 9.07, N 7.91.

Synthesis of 4,4-dimethylmorpholinium chloride (NDMMCl)

A column was packed with Purolite SGA550OH ion exchange resin with the mole ratio of 2:1 resin to NDMMI. NDMMI was synthesized according to the reported procedure [1]. NDMMI was dissolved in methanol to the final concentration of 0.1 M and passed through the column with the flow rate of 1 drop per second. Later the column was washed with 400-500 ml of methanol and the solution was neutralized with HCl 1 M. Subsequent removal of the solvent using rotatory evaporation and later vacuum drying gave white hygroscopic salt.

White hygroscopic solid. Elemental analysis for $C_6H_{14}NOCl$ calc. (%): C 47.53, H 9.31, N 9.24. Found: C 47.04, H 9.23, N 9.08.

Synthesis of 4-ethyl-4-methylmorpholinium chloride (EMMorCl)

A column was packed with Purolite SGA550OH ion exchange resin with the mole ratio of 2:1 resin to EMMorBr. EMMorBr was dissolved in methanol to the final concentration of 0.1 M and passed through the column with the flow rate of 1 drop per second. Later the column was washed with 400-500 ml of methanol and the solution was neutralized with HCl 1 M. Subsequent removal of the solvent using rotatory evaporation and later vacuum drying gave white hygroscopic salt.

White hygroscopic solid. Elemental analysis for $C_7H_{16}NOCl$ calc. (%): C 50.75, H 9.74, N 8.46. Found: C 50.32, H 9.60, N 8.50.

Synthesis of 4-methyl-4-propylmorpholinium chloride (MPMorCl)

A column was packed with Amberlite-IR410 Cl ion exchange resin with the mole ratio of 2:1 resin to MPMorBr. MPMorBr was dissolved in methanol to the final concentration of 0.1 M and passed through the column with the flow rate of 1 drop per second. Later the column was washed with 400-500 ml of methanol and subsequent removal of the solvent using rotatory evaporation and later vacuum drying gave white hygroscopic salt.

White hygroscopic solid. Elemental analysis for $C_8H_{18}NOCl$ calc. (%): C 53.47, H 10.10, N 7.80. Found: C 52.70, H 9.94, N 7.71.

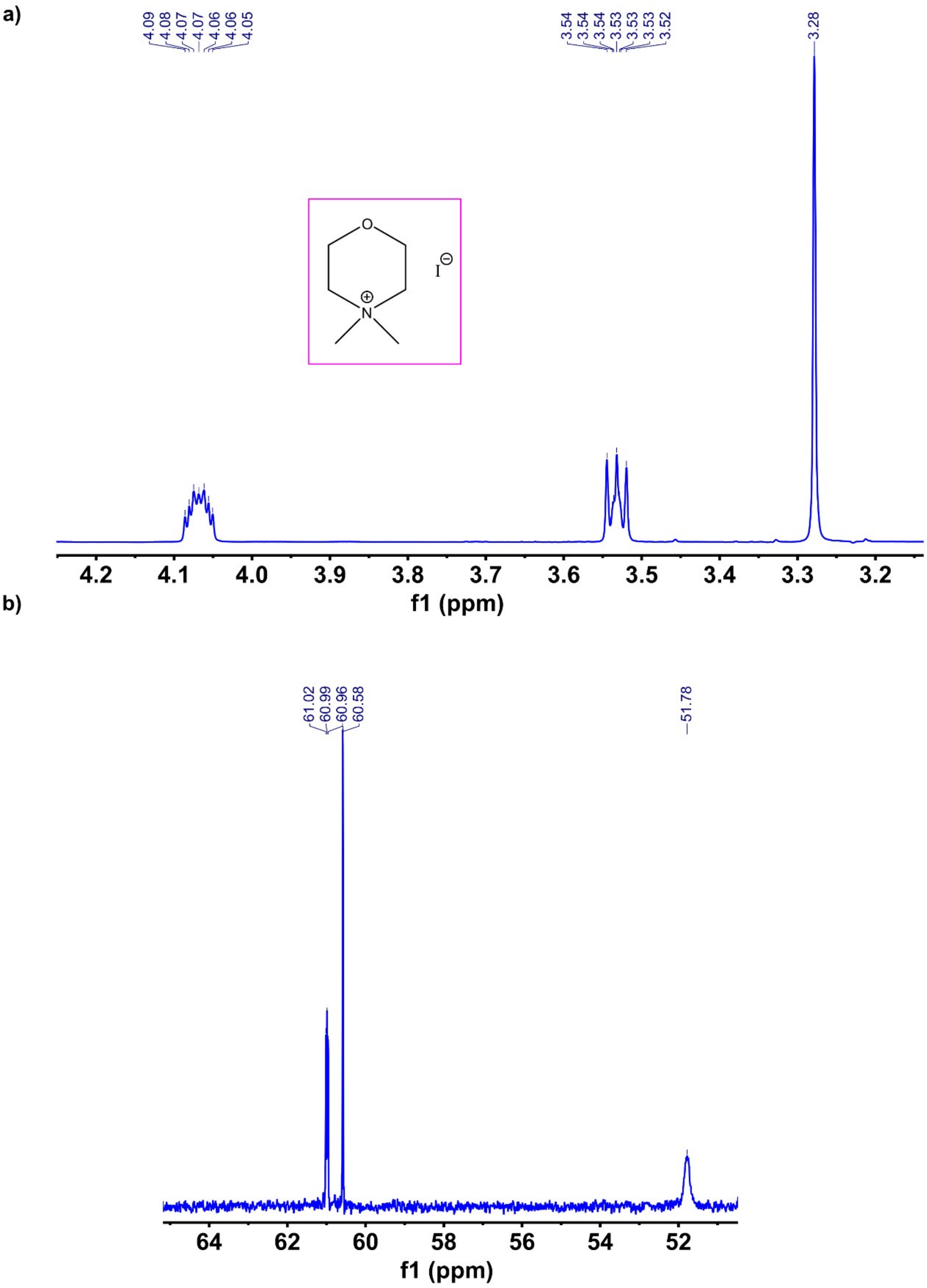

Figure 1. a) ^{1}H NMR b) ^{13}C NMR of NDMMI in D_2O

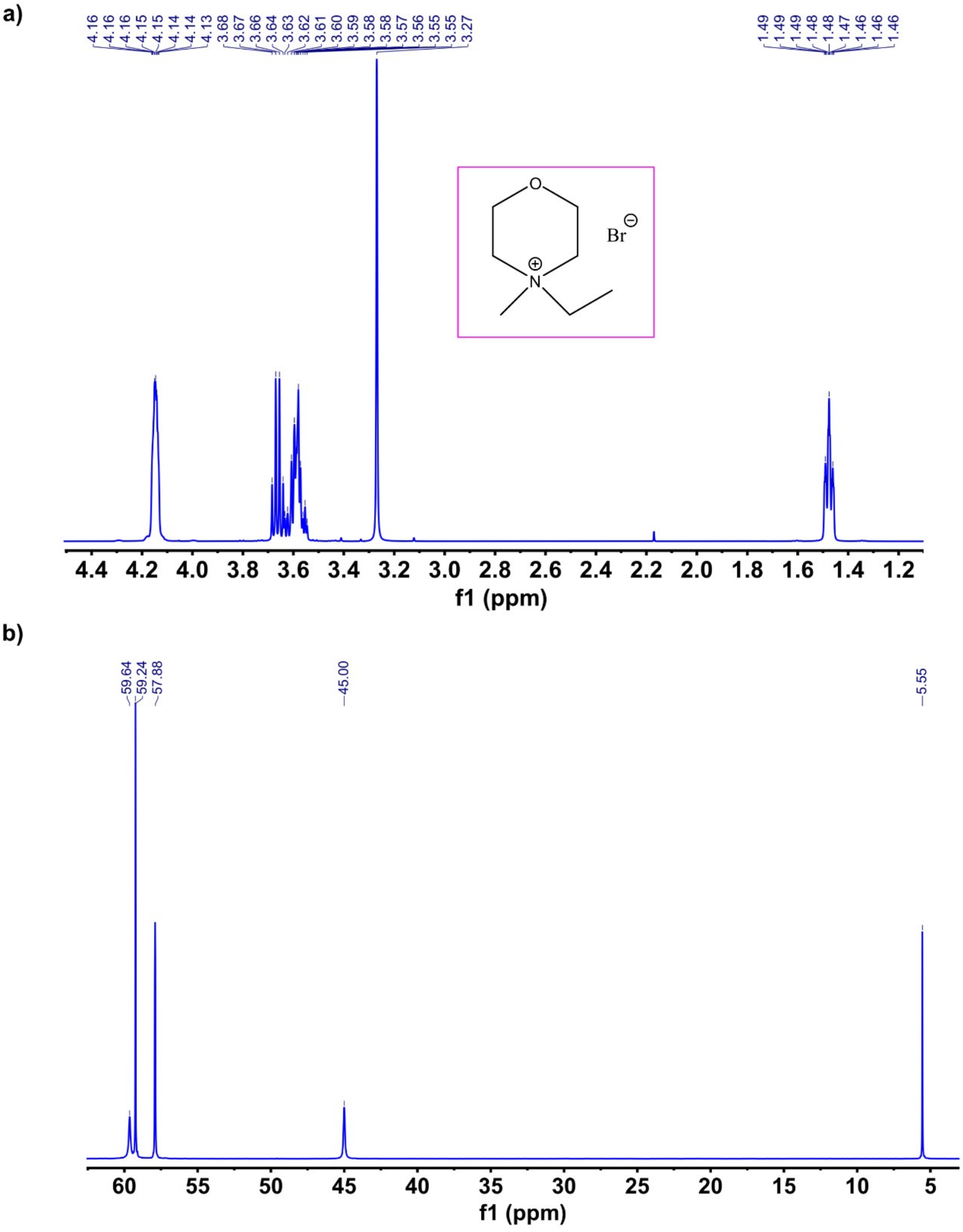

Figure 2. a) ^{1}H NMR b) ^{13}C NMR of EMMorBr in D_2O

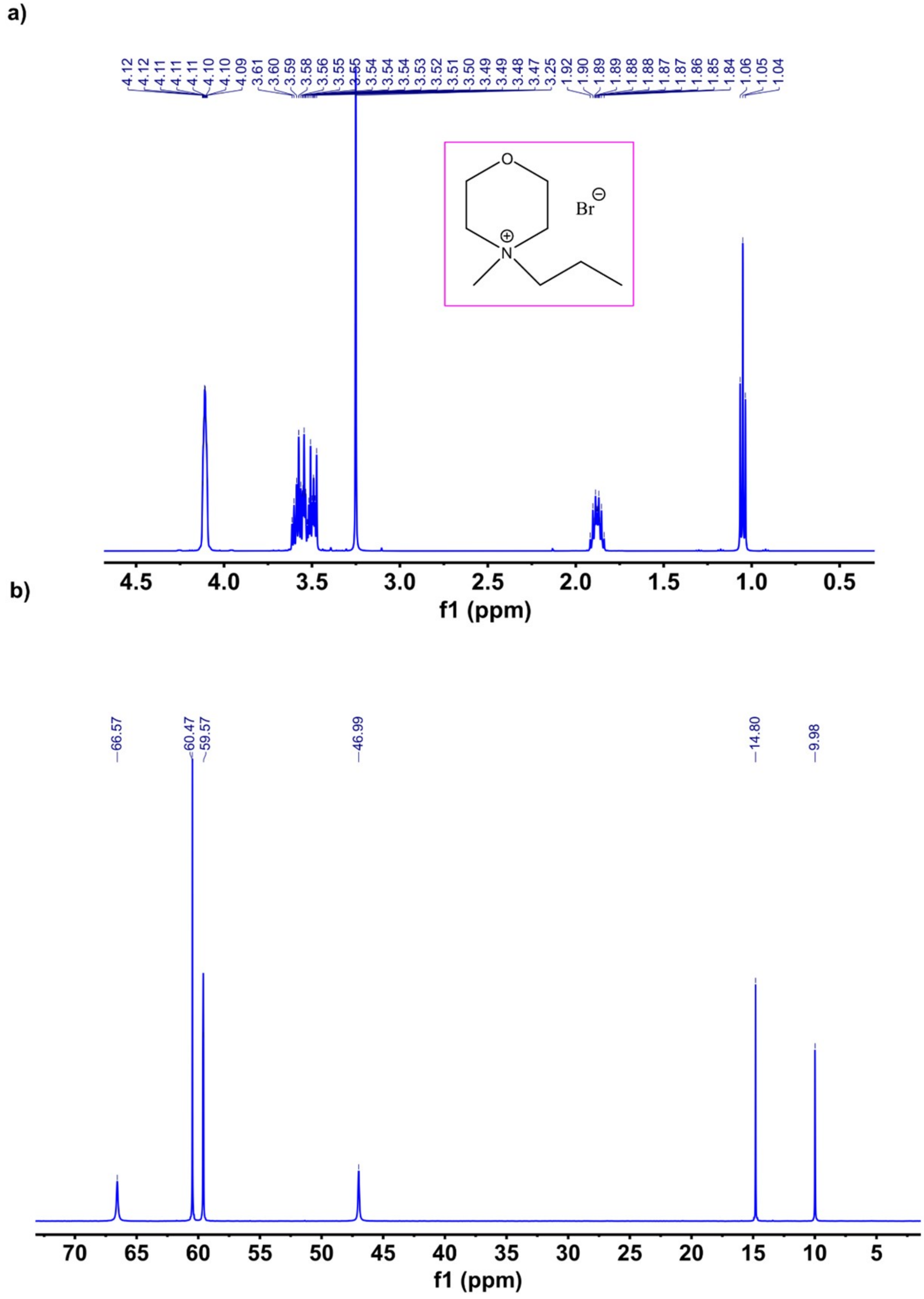

Figure 3. a) ^{1}H NMR b) ^{13}C NMR of MPMorBr in D_2O

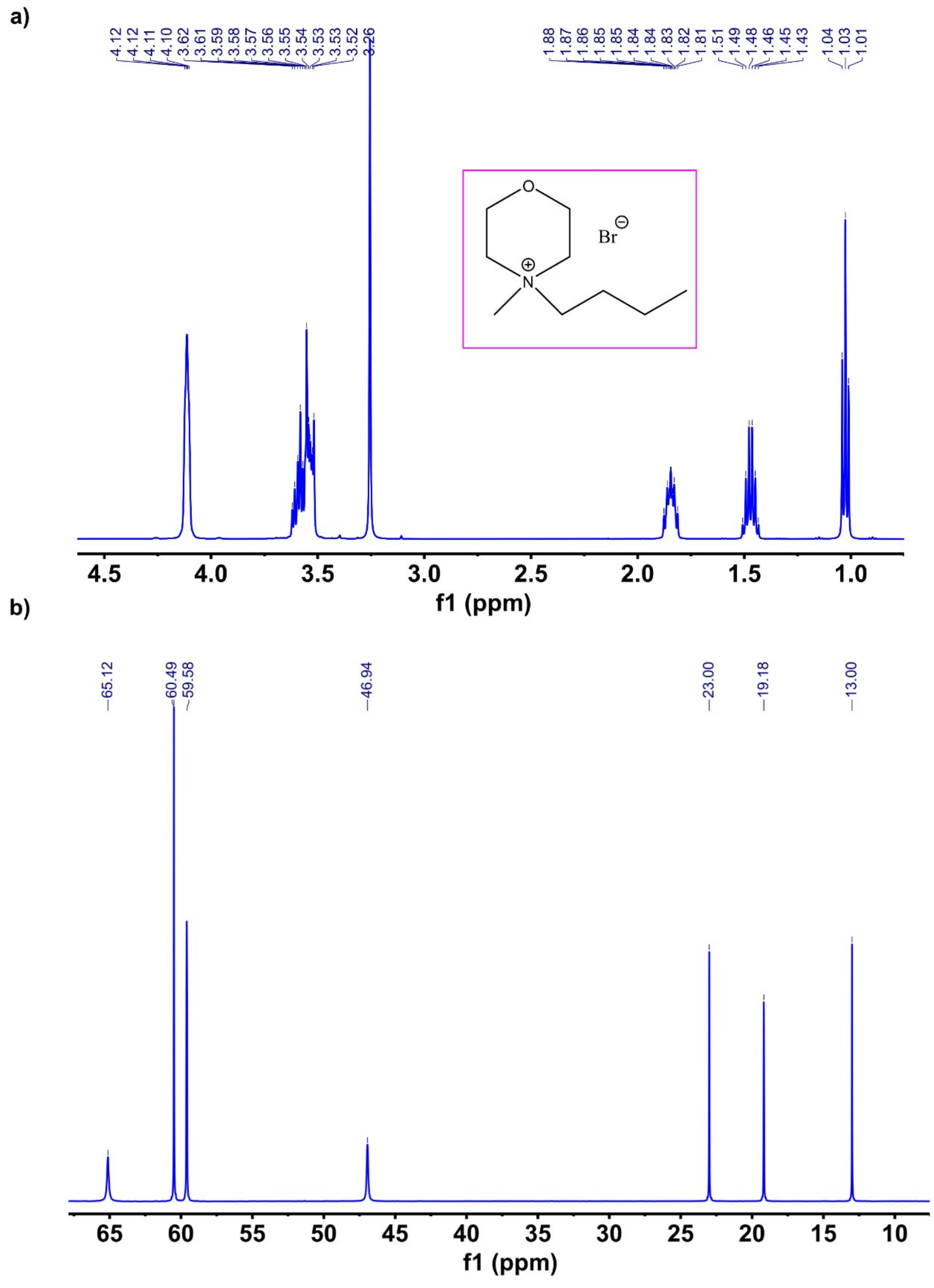

Figure 4. a) ^{1}H NMR b) ^{13}C NMR of BMMorBr in D_2O

a)

b)

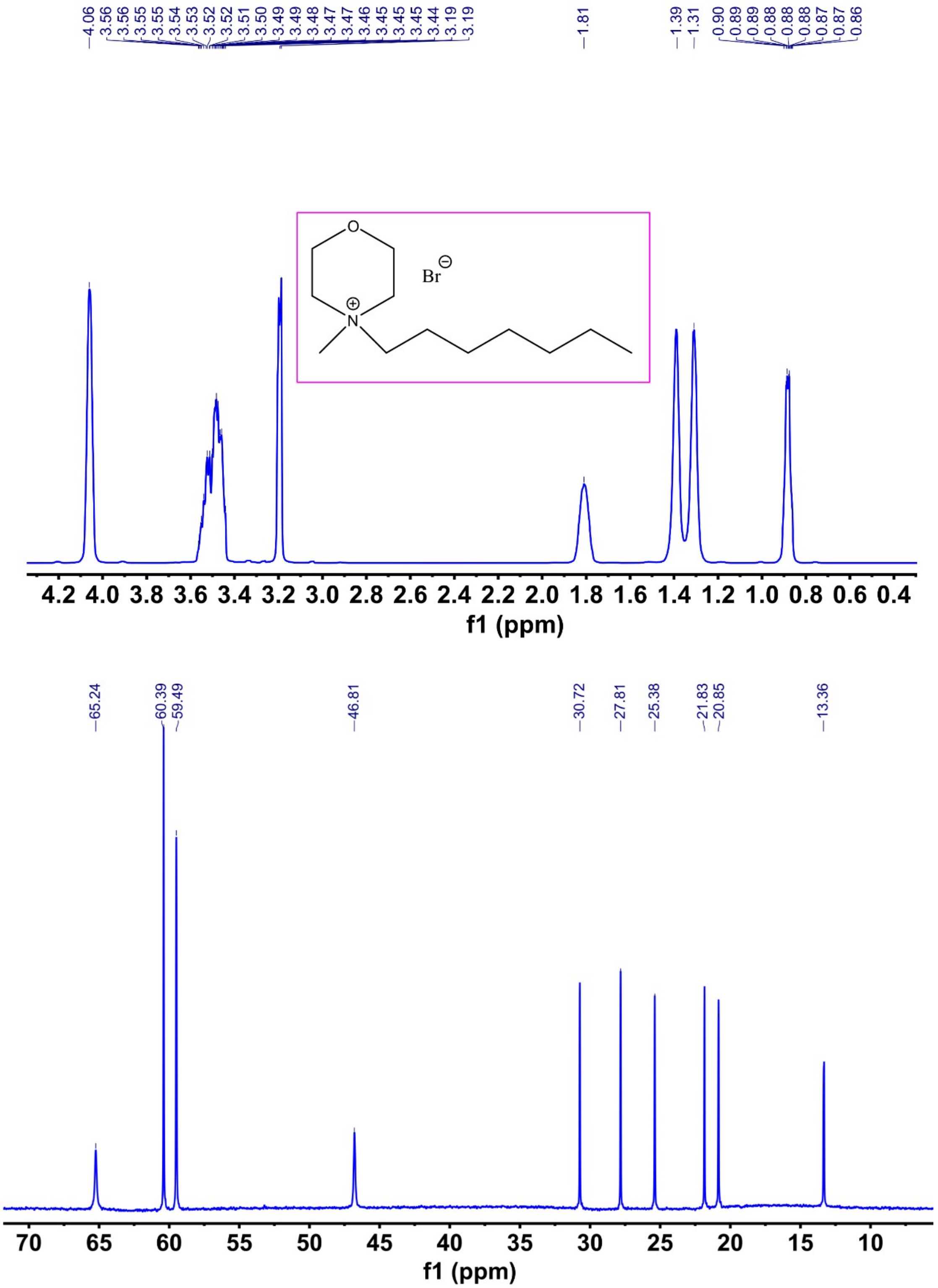

Figure 5. a) ^{1}H NMR b) ^{13}C NMR of HMMorBr in D_2O

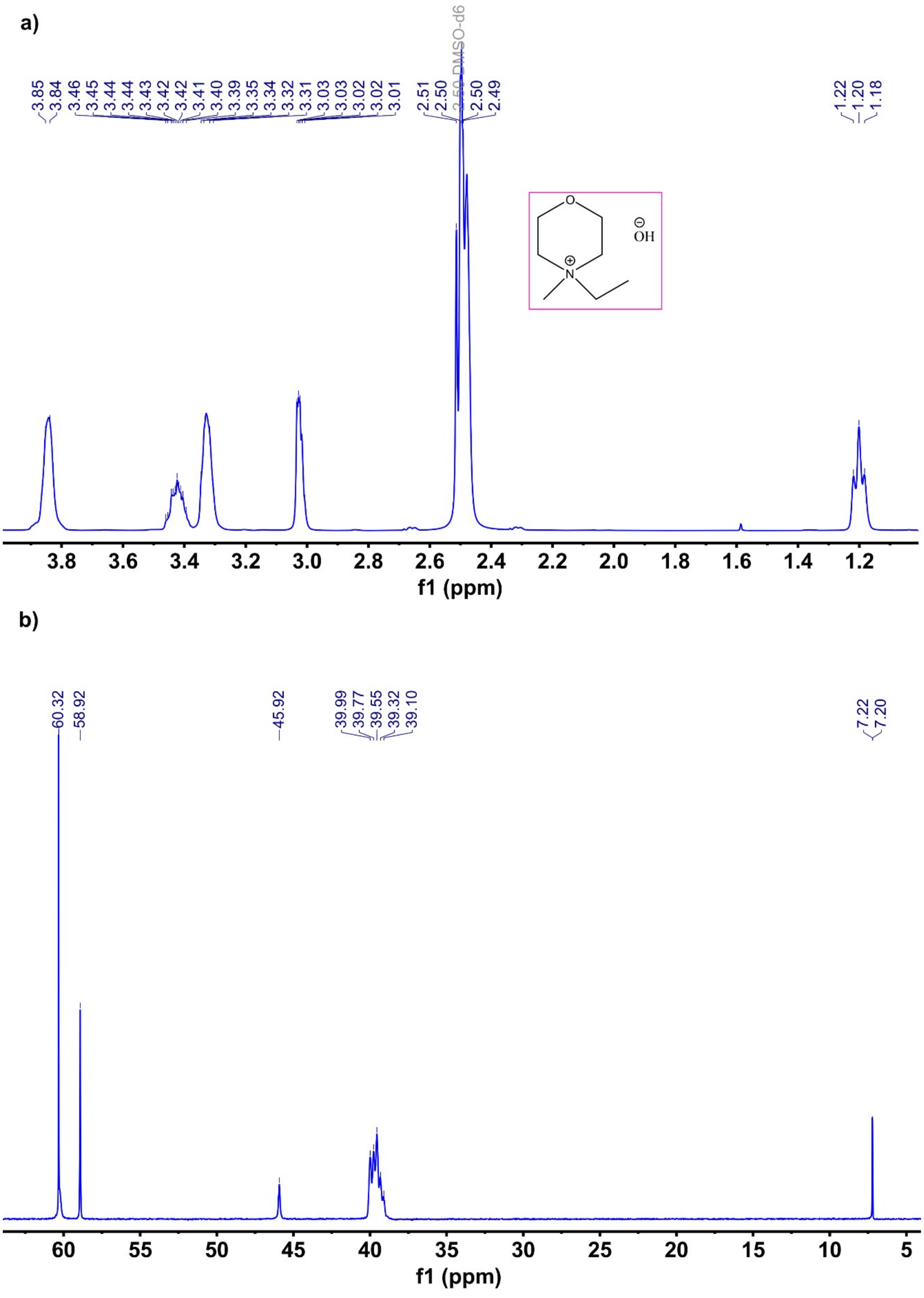

Figure 6. a) ^{1}H NMR b) ^{13}C NMR of EMMorOH(aq) in DMSO-d_6

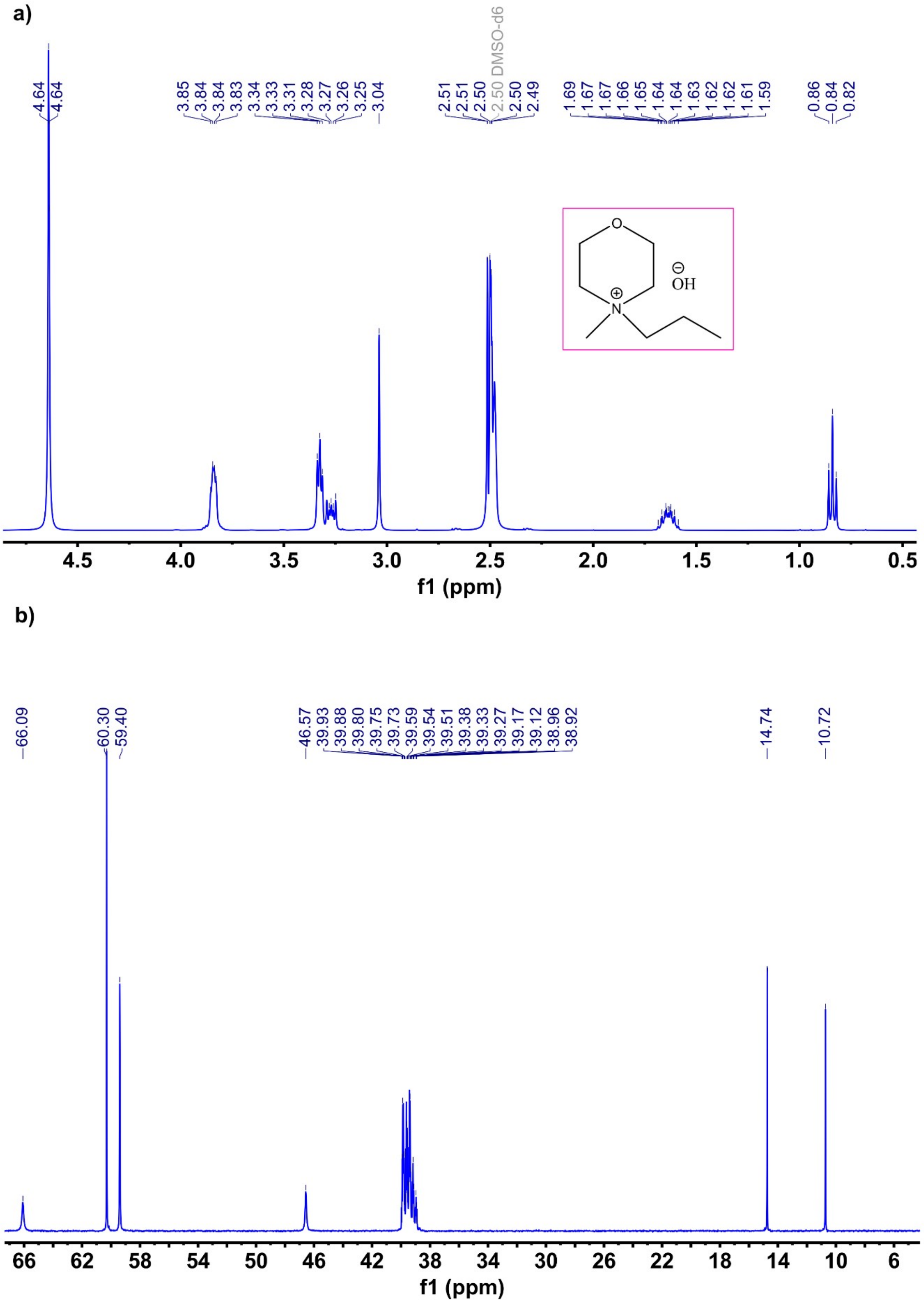

Figure 7. a) ^{1}H NMR b) ^{13}C NMR of MPMorOH(aq) in DMSO-d_6

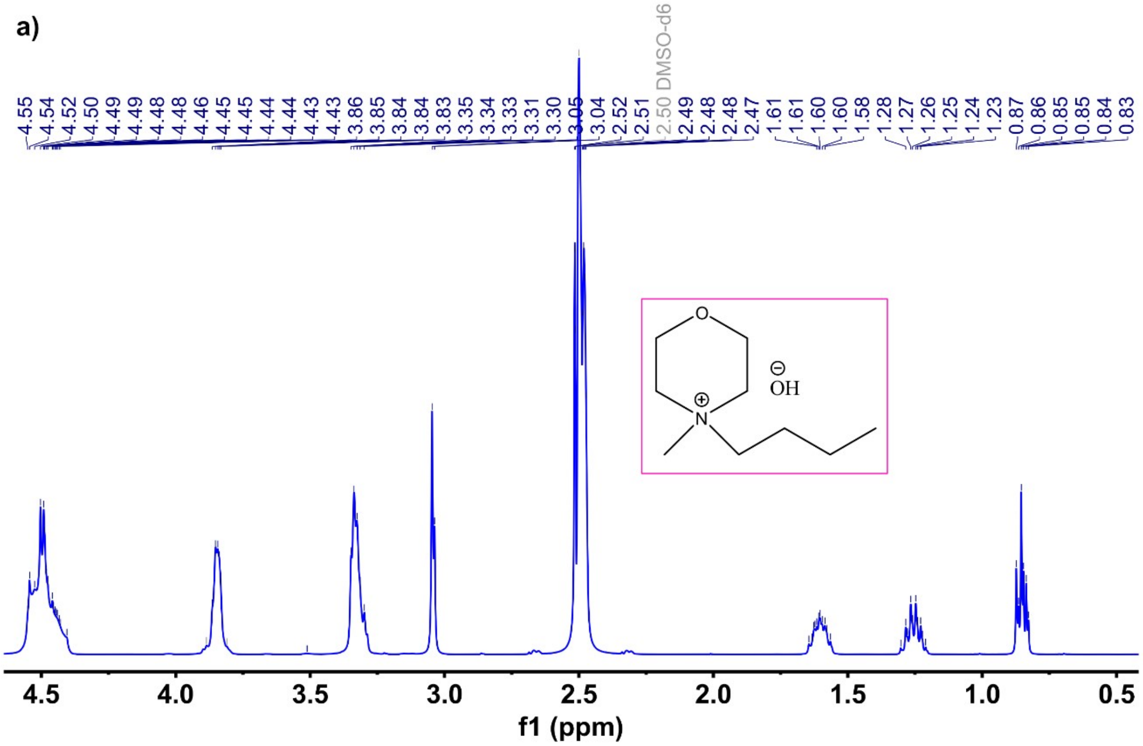

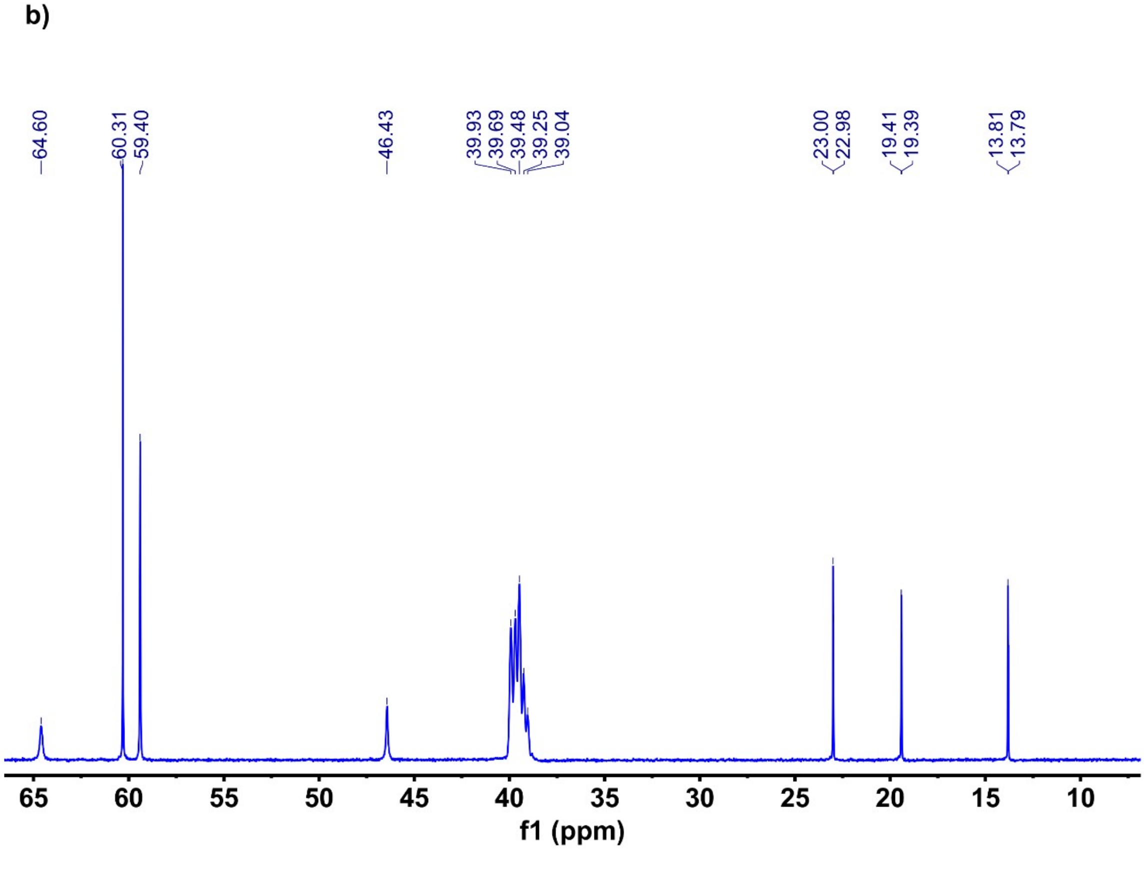

Figure 8. a) ^{1}H NMR b) ^{13}C NMR of BMMorOH(aq) in DMSO-d_6

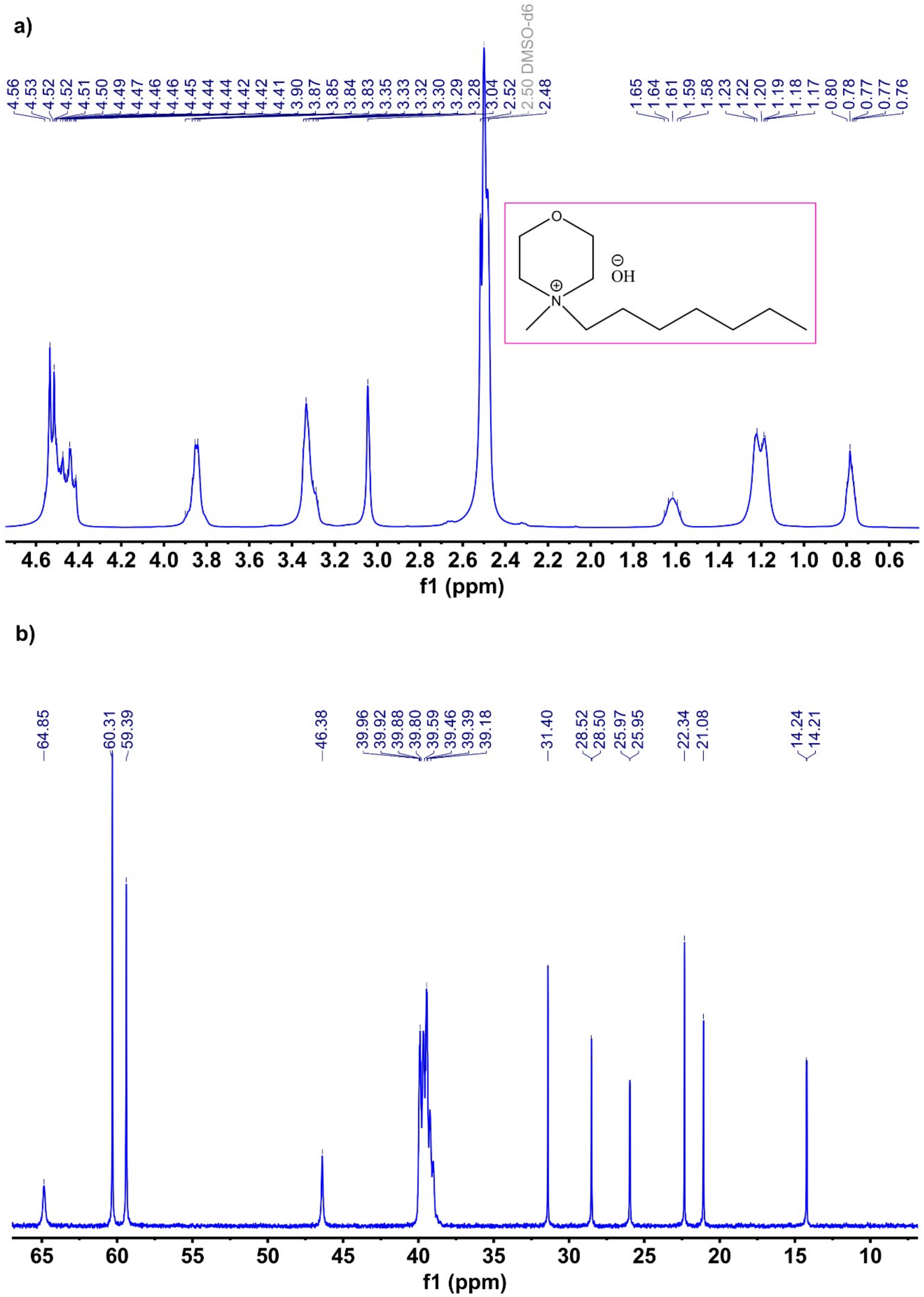

Figure 9. a) ^{1}H NMR b) ^{13}C NMR of HMMorOH(aq) in DMSO-d_6

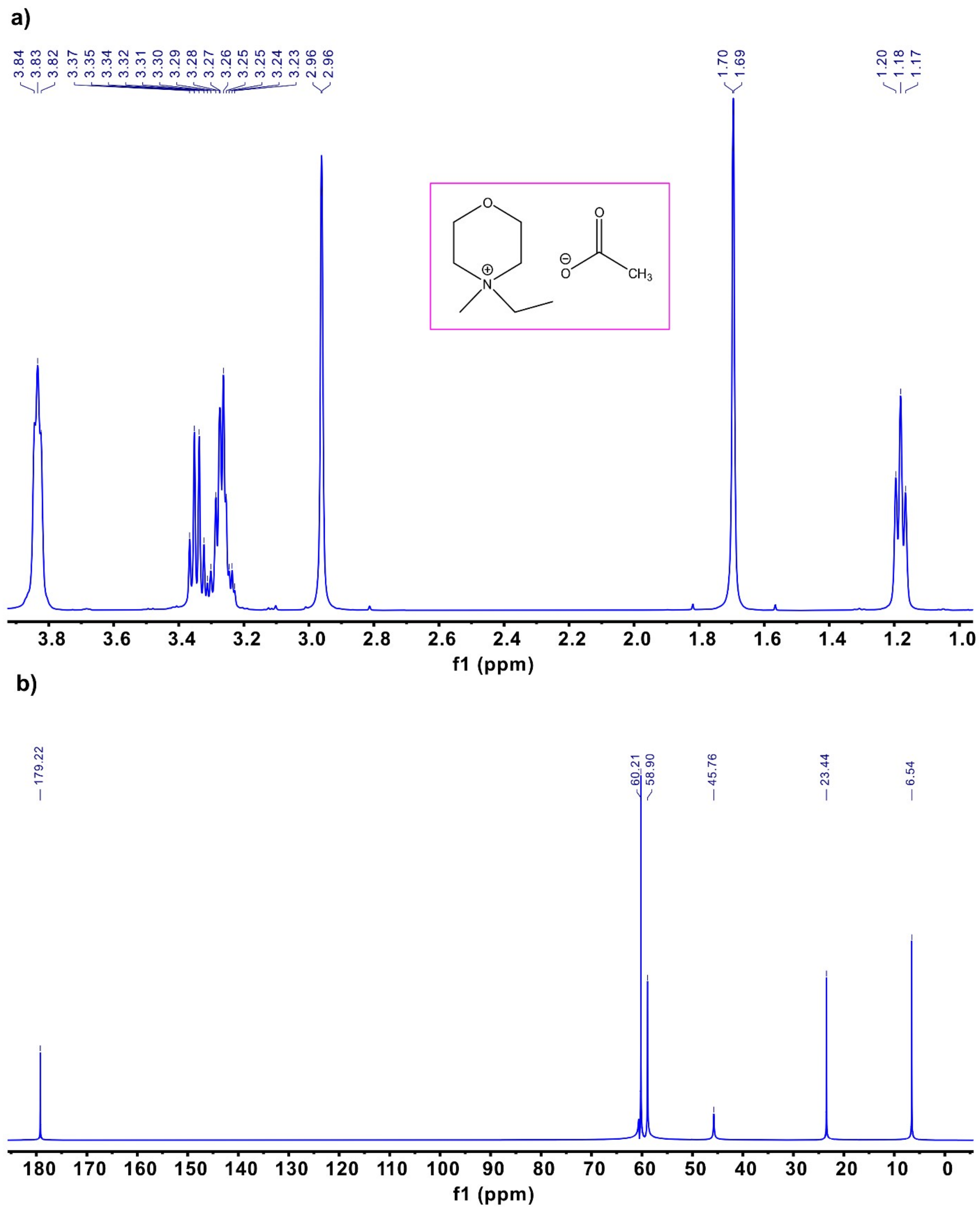

Figure 10. a) ^{1}H NMR b) ^{13}C NMR of EMMorOAc in D_2O

a)

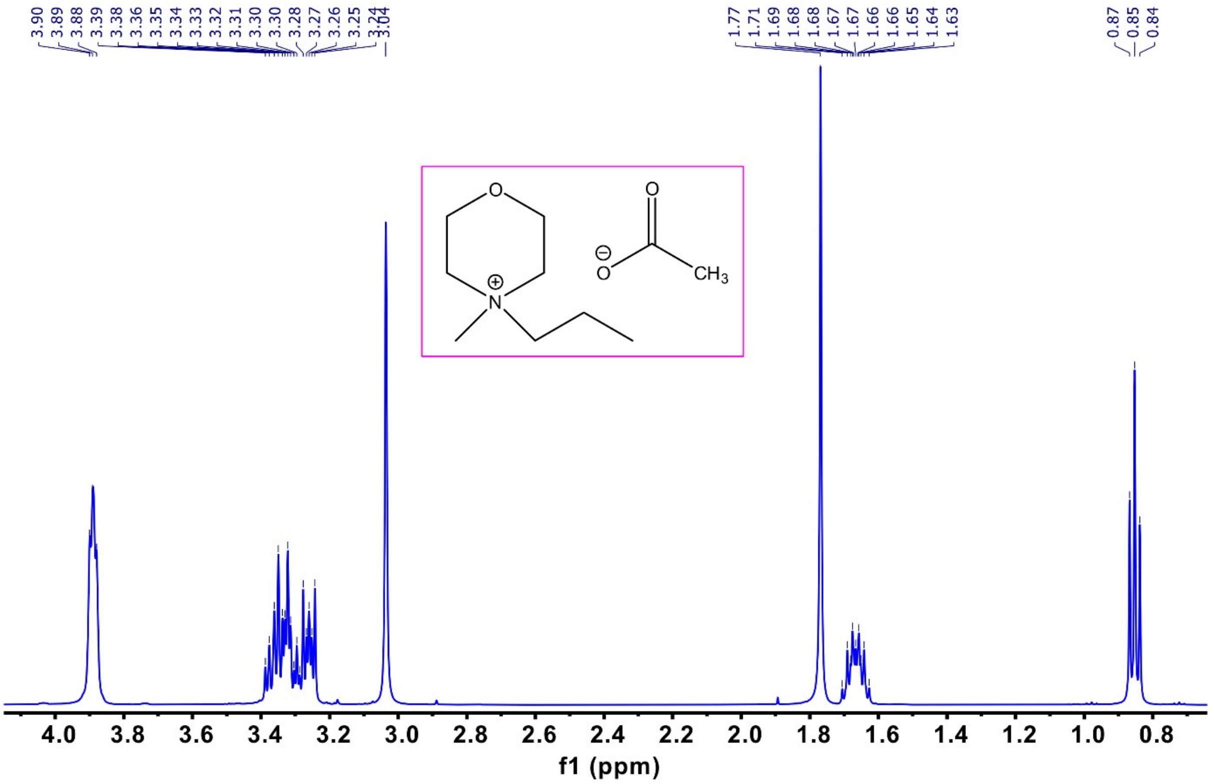

b)

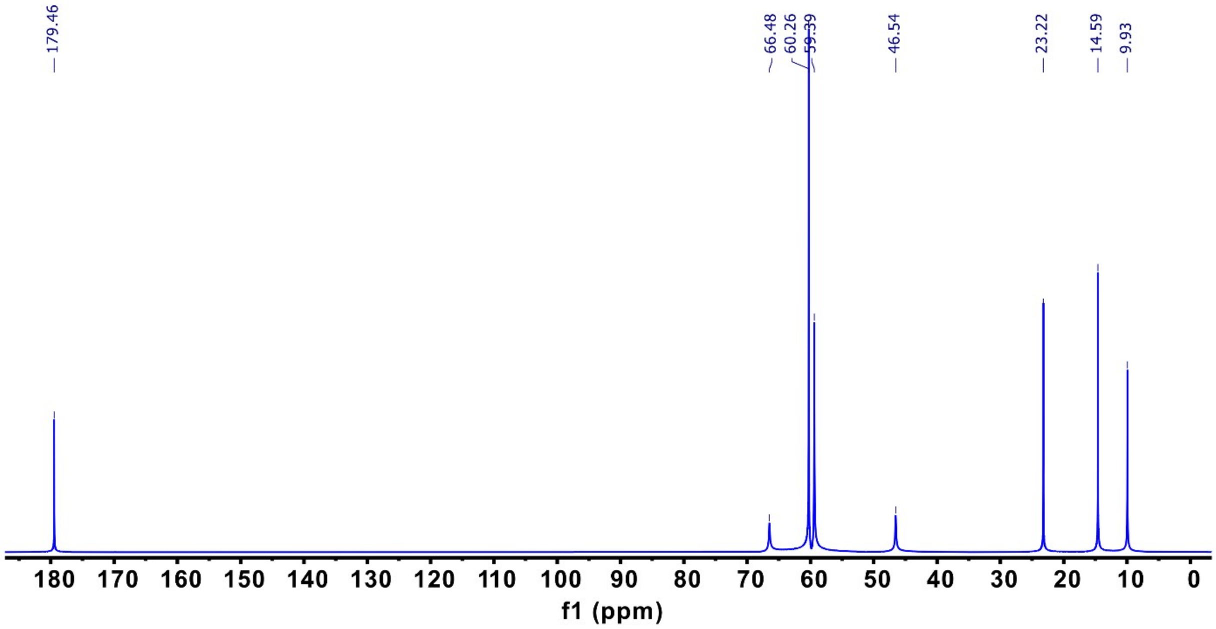

Figure 11. a) ^{1}H NMR b) ^{13}C NMR of MPMorOAc in D_2O

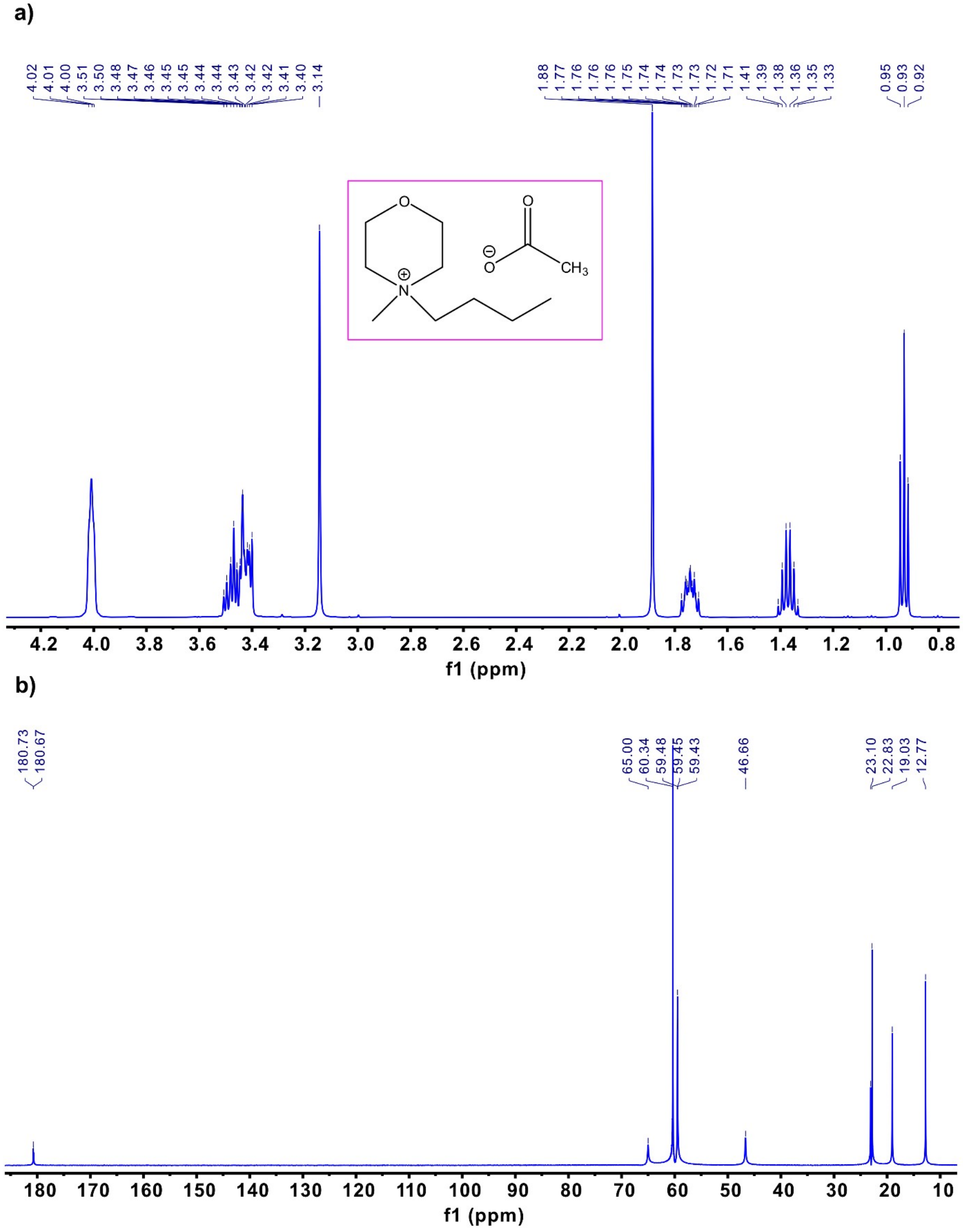

Figure 12. a) ^{1}H NMR b) ^{13}C NMR of BMMorOAc in D_2O

a)

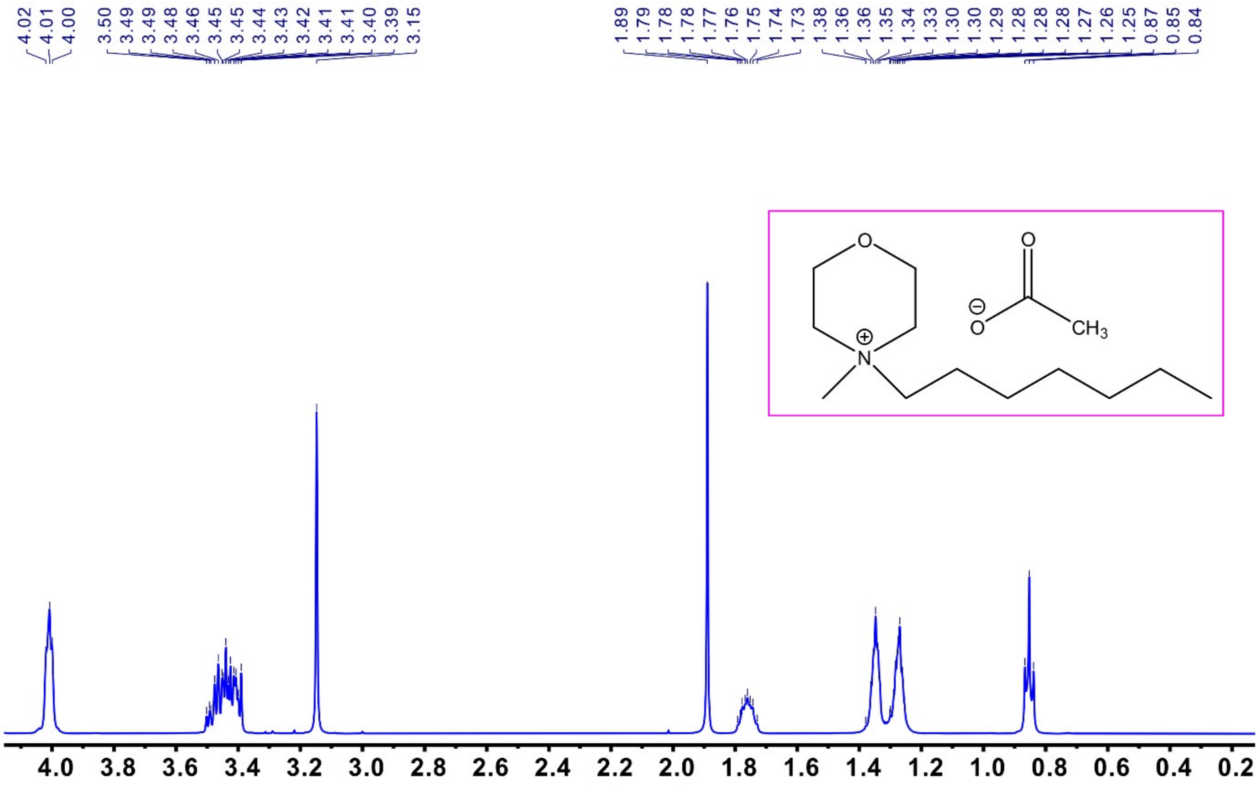

b)

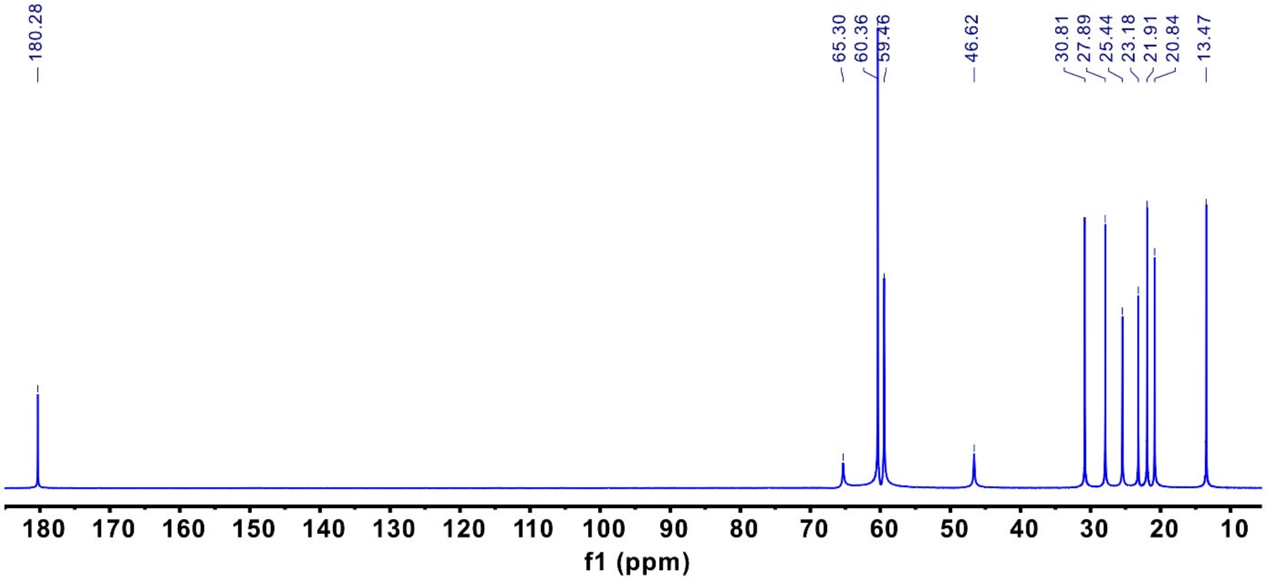

Figure 13. a) ^{1}H NMR b) ^{13}C NMR of HMMorOAc in D_2O

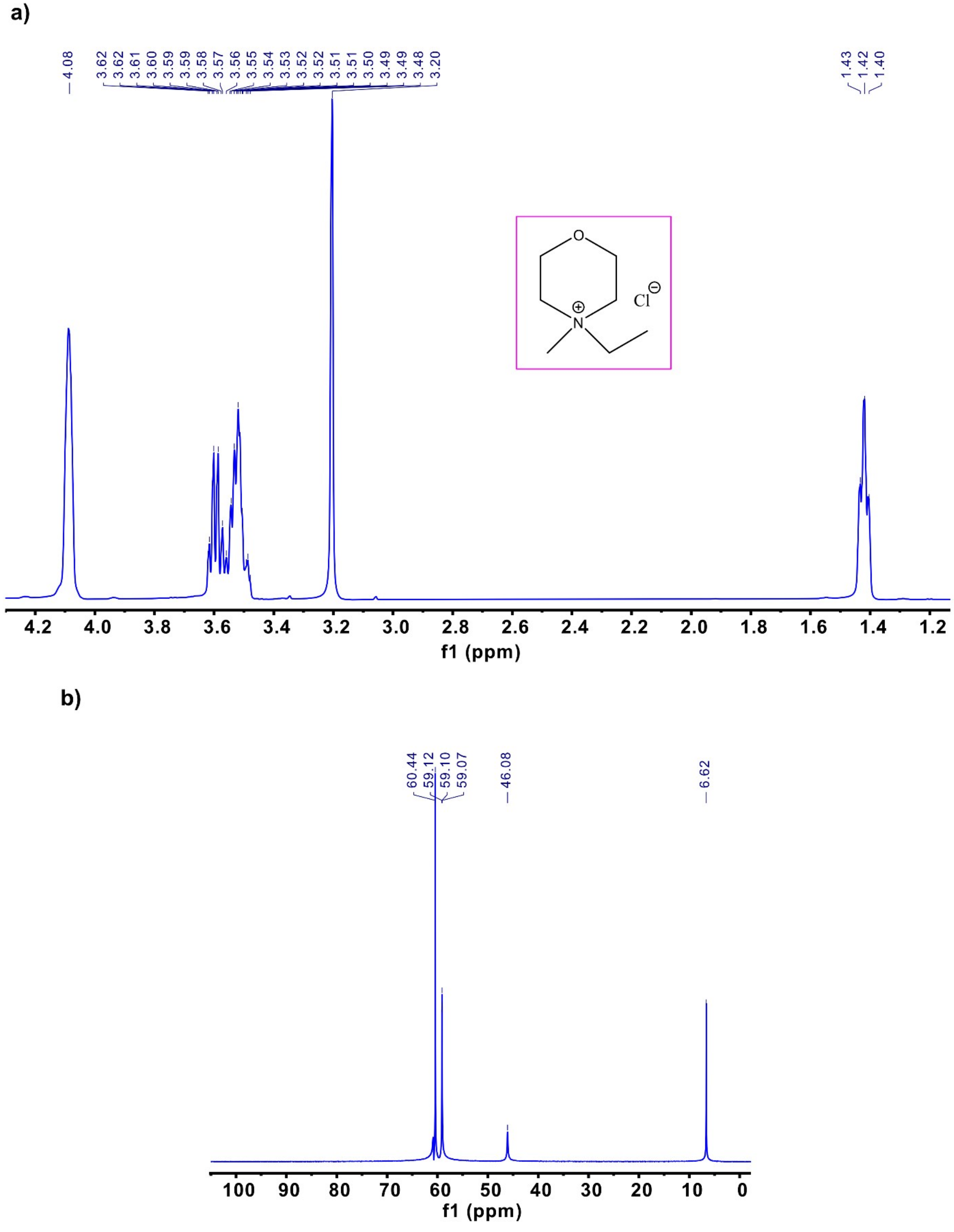

Figure 14. a) ^1H NMR b) ^{13}C NMR of EMMorCl in D_2O

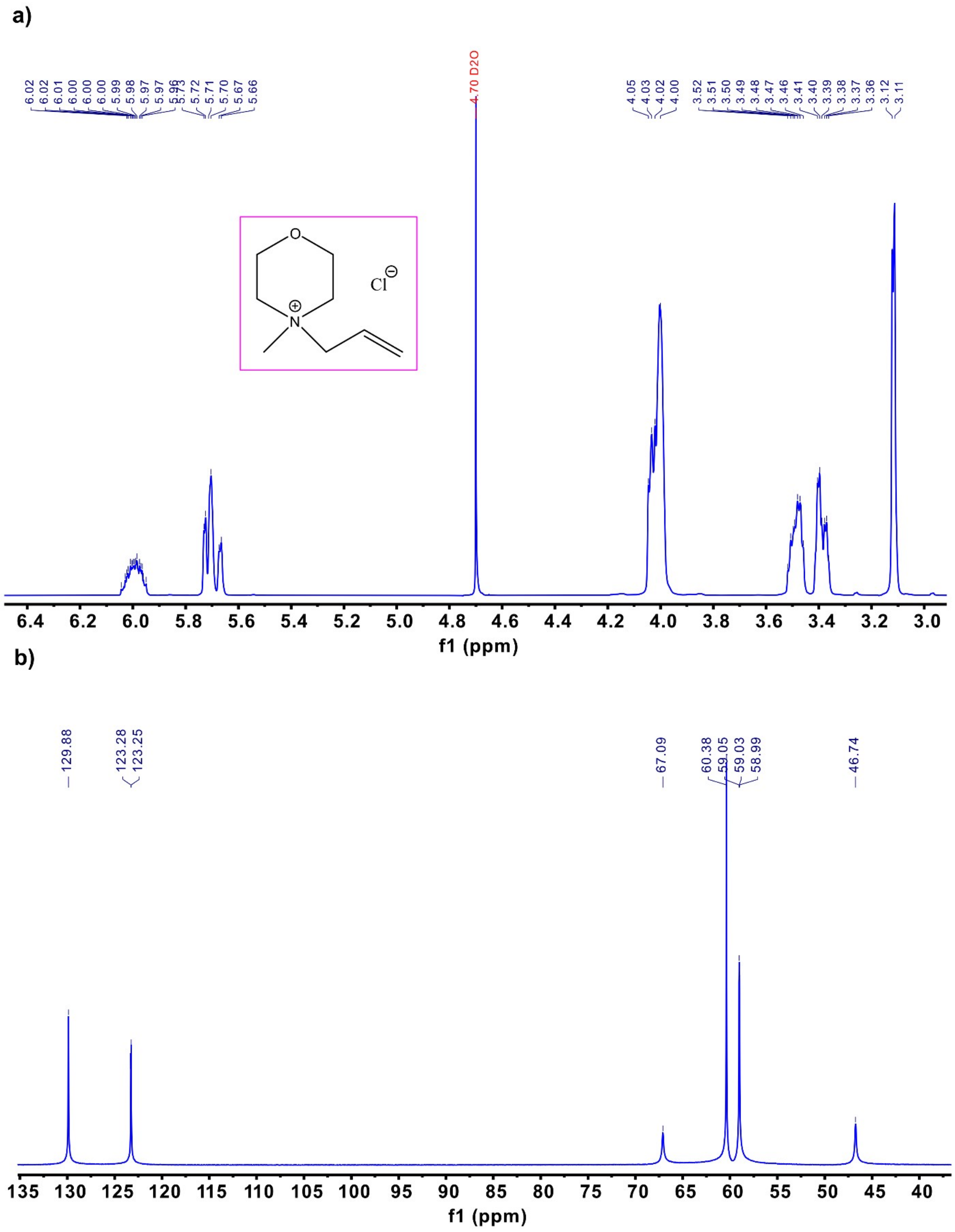

Figure 15. a) ^{1}H NMR b) ^{13}C NMR of AMMorCl in D_2O

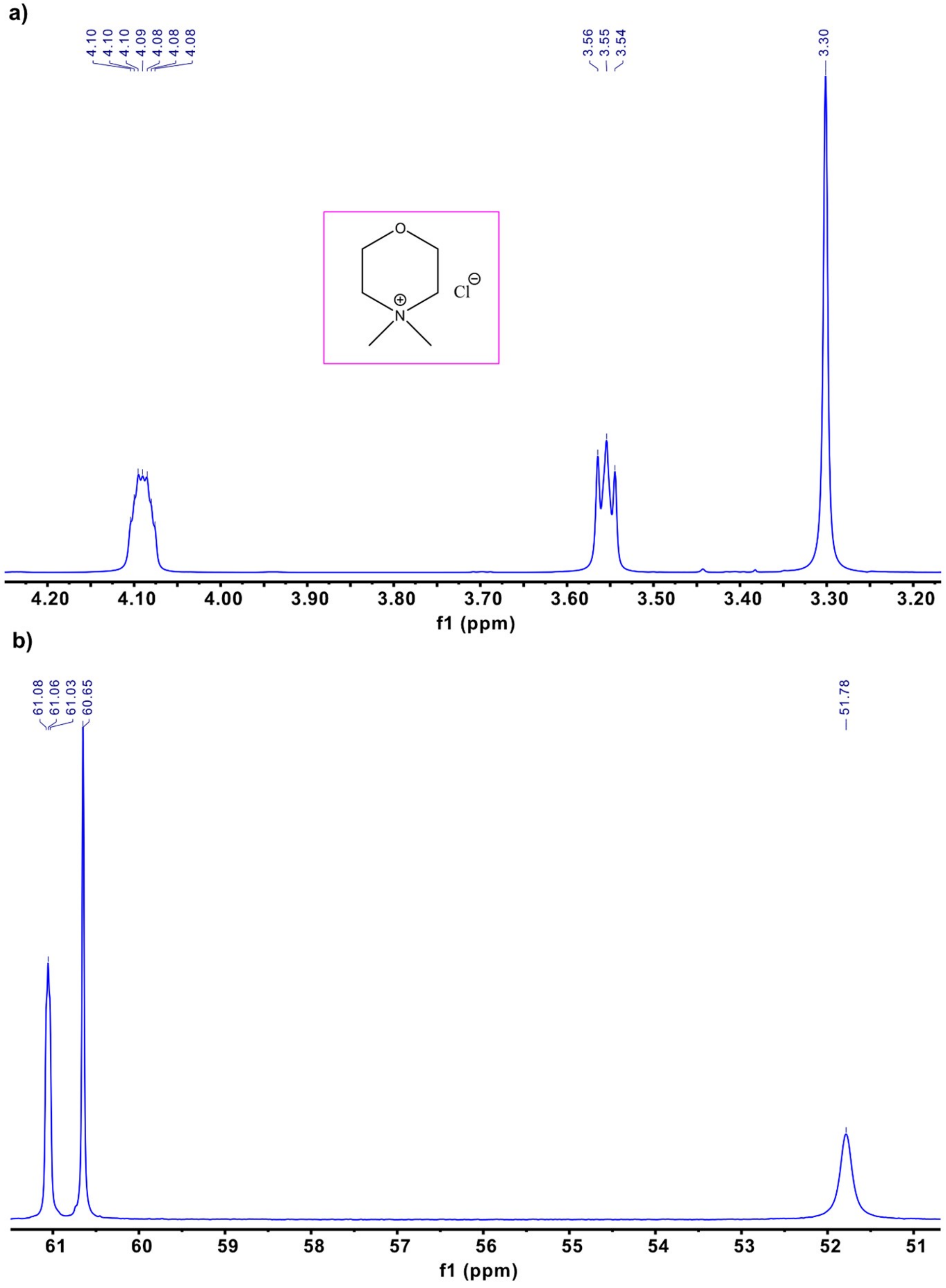

Figure 16. a) ^{1}H NMR b) ^{13}C NMR of NDMMCl in D_2O

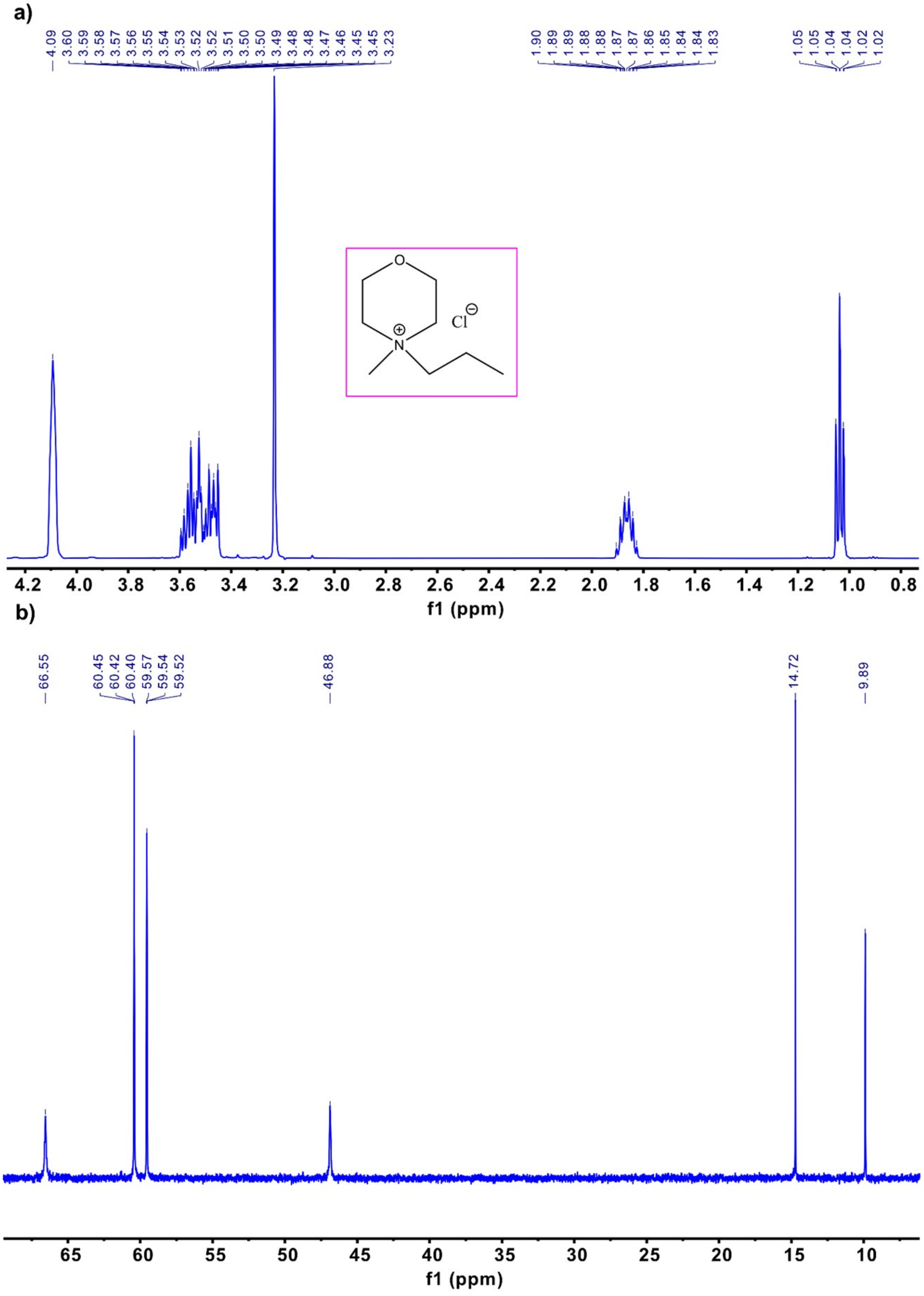

Figure 17. a) ^{1}H NMR b) ^{13}C NMR of MPMorCl in D_2O

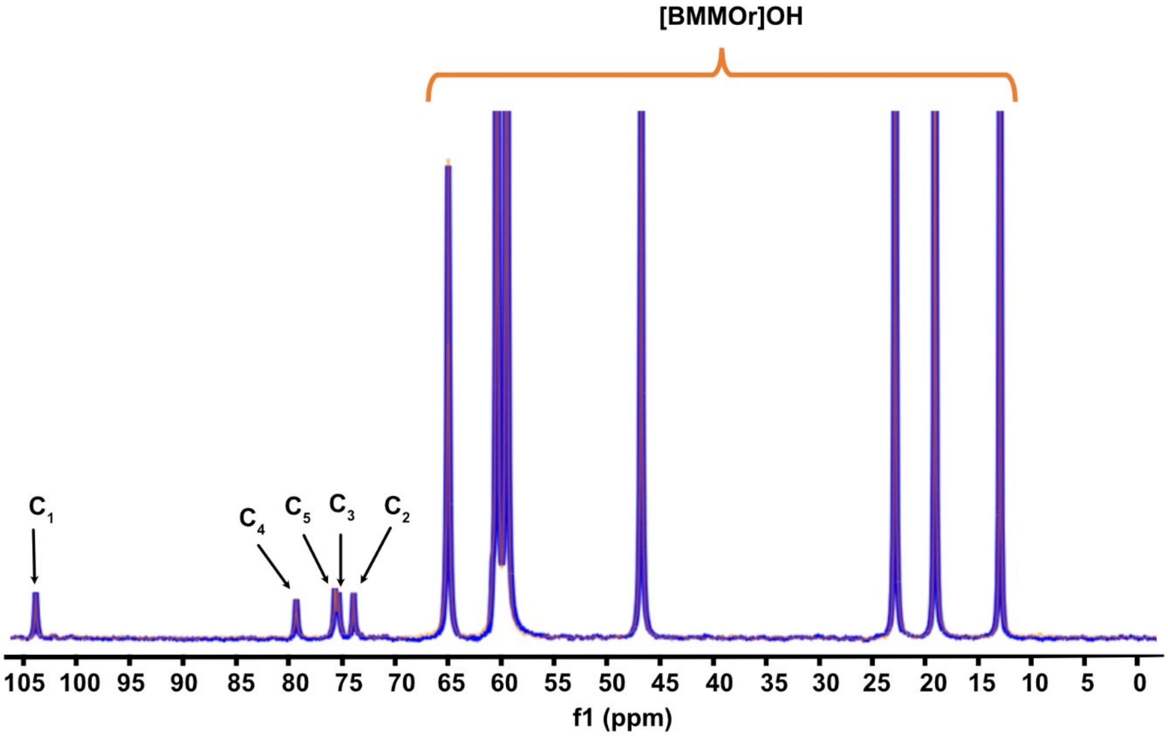

Figure 18. ^{13}C NMR measurements of freshly dissolved MCC 4 wt% in BMMorOH(aq) (orange) and an aged solution for 22 h post dissolution (blue). Due to perfect overlap of the spectra the orange spectrum can not be seen.

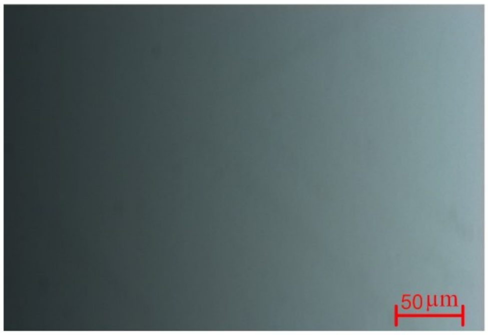

Figure 19. Optical microscopy image of completely dissolved cellulose

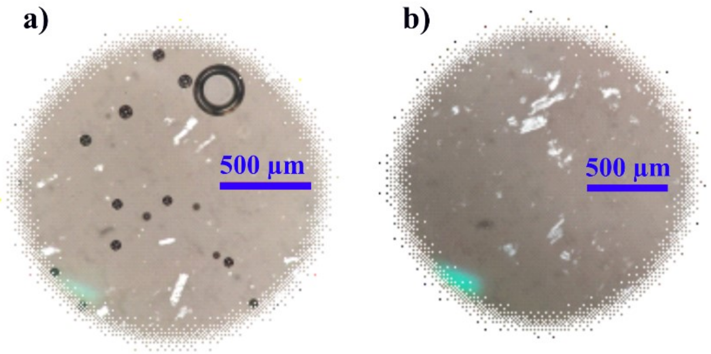

Figure 20. Optical microscopy images of undissolved cellulose in a) HMMorOAc/DMSO b) BMMorOAc/DMSO

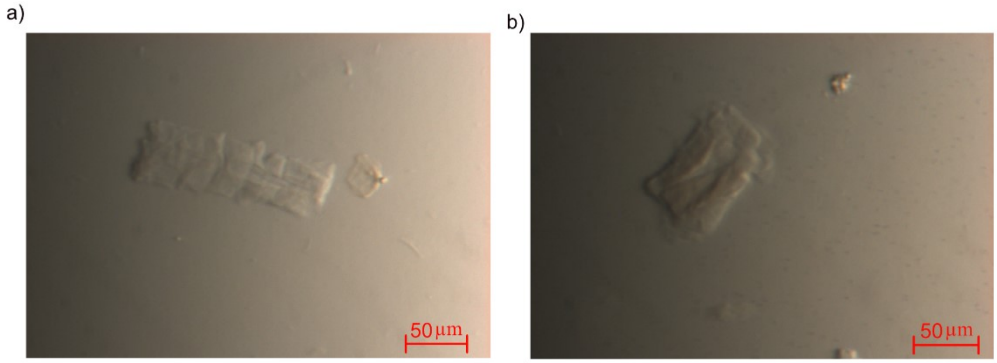

Figure 21. Optical microscopy images of undissolved cellulose in a) MPMorOH(aq) b) BMMorOH(aq)

Table 1. Detailed SEC analysis values

Sample name	Mw (kg/mol)	Mp (kg/mol)	Mn (kg/mol)	Mv (kg/mol)	PD
Reference Cellulose	91.2	52.9	19.6	239.7	4.6
Cellulose in EMMorOAc/DMSO	84.9	51.7	18	219.3	4.7
Cellulose in BMMorOAc/DMSO	84.8	52.9	18.6	215.9	4.6
Cellulose in MPMorOH	84.5	50.5	19.4	209.7	4.4
Cellulose in BMMorOH	82.1	50.5	17.3	203.2	4.7

References

1 S. Naserifar, B. Swensson, D. Bernin and M. Hasani, *Eur. Polym. J.*, 2021, **161**, 110822.
